# Landscape‐variability of the carbon balance across managed boreal forests

**DOI:** 10.1111/gcb.16534

**Published:** 2022-12-04

**Authors:** Matthias Peichl, Eduardo Martínez‐García, Johan E. S. Fransson, Jörgen Wallerman, Hjalmar Laudon, Tomas Lundmark, Mats B. Nilsson

**Affiliations:** ^1^ Department of Forest Ecology and Management Swedish University of Agricultural Sciences (SLU) Umeå Sweden; ^2^ Department of Forest Resource Management Swedish University of Agricultural Sciences (SLU) Umeå Sweden; ^3^ Department of Forestry and Wood Technology Linnaeus University Växjö Sweden

**Keywords:** boreal forest landscape, carbon sequestration, climate change mitigation, forest management, heterotrophic respiration, net primary production, rotation‐forestry

## Abstract

Boreal forests are important global carbon (C) sinks and, therefore, considered as a key element in climate change mitigation policies. However, their actual C sink strength is uncertain and under debate, particularly for the actively managed forests in the boreal regions of Fennoscandia. In this study, we use an extensive set of biometric‐ and chamber‐based C flux data collected in 50 forest stands (ranging from 5 to 211 years) over 3 years (2016–2018) with the aim to explore the variations of the annual net ecosystem production (NEP; i.e., the ecosystem C balance) across a 68 km^2^ managed boreal forest landscape in northern Sweden. Our results demonstrate that net primary production rather than heterotrophic respiration regulated the spatio‐temporal variations of NEP across the heterogeneous mosaic of the managed boreal forest landscape. We further find divergent successional patterns of NEP in our managed forests relative to naturally regenerating boreal forests, including (i) a fast recovery of the C sink function within the first decade after harvest due to the rapid establishment of a productive understory layer and (ii) a sustained C sink in old stands (131–211 years). We estimate that the rotation period for optimum C sequestration extends to 138 years, which over multiple rotations results in a long‐term C sequestration rate of 86.5 t C ha^−1^ per rotation. Our study highlights the potential of forest management to maximize C sequestration of boreal forest landscapes and associate climate change mitigation effects by developing strategies that optimize tree biomass production rather than heterotrophic soil C emissions.

## Graphical abstract

The carbon (C) balance of actively managed boreal forests has remained uncertain. Using biometric‐ and chamber‐based C flux data collected in 50 forest stands, we explored variations of net ecosystem production (NEP) across a managed forest landscape in northern Sweden. We find that net primary production rather than heterotrophic respiration regulated its spatio‐temporal variations. Divergent successional patterns relative to naturally regenerating boreal forests include a fast C sink recovery within the first decade and a sustained C sink in old stands. Our study highlights the potential of boreal forest management to maximize C sequestration by optimizing tree biomass production.

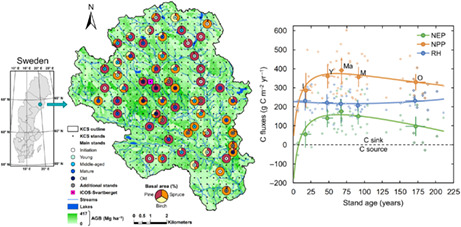

## 1 INTRODUCTION

Boreal forests cover 10%–15% of the terrestrial land area and store about one third of the global forest carbon (C) pool (Astrup et al., 2018; Pan et al., 2011). Due to their large capacity for sequestering carbon dioxide (CO_2_) from the atmosphere into biomass and soil (Myneni et al., 2001), these ecosystems are considered as key element in national and international policy frameworks for mitigating climate change (Lemprière et al., 2013; Lundmark et al., 2014; UNFCC, 2016). To ensure accurate accounting in these strategies, we require a well‐founded empirical database on the boreal forest C sink strength (Bellassen & Luyssaert, 2014). Furthermore, boreal forests experience the most rapid changes in climatic conditions (Gauthier et al., 2015), which calls for a detailed understanding of future forest C cycle‐climate feedback in the boreal biome.

A key limitation to these ambitions is that the boreal forest C cycle has remained understudied relative to other, e.g., temperate regions (Luyssaert et al., 2007; Xu et al., 2014). Moreover, there is a bias toward studies in boreal Canada and Russia, where the boreal forest succession is often driven by natural disturbance regimes, such as wildfires, insect outbreaks and wind throws (Amiro et al., 2010; Bond‐Lamberty et al., 2004; Virkkala et al., 2022). In comparison, our knowledge on the magnitudes and variations of the forest C balance of actively managed landscapes occurring within the boreal regions of Fennoscandia is limited (Bellassen & Luyssaert, 2014). This part of the boreal forest biome is predominately managed by rotation forestry, which usually consists of clear‐cutting followed by soil scarification, planting or natural regeneration using seed trees, and several thinning operations until the subsequent clear‐cut harvest (Felton et al., 2020). At the landscape level, this system creates a spatial mosaic resembling the age class distribution of different even‐aged stands, which are characterized by distinct C cycles. Management activities (e.g., fertilization, thinning, drainage, and soil scarification) further enhance this spatial heterogeneity through effects on tree growth rates, soil C dynamics, and woody debris pools (Noormets et al., 2015; Xu et al., 2014). Due to data scarcity and associated uncertainties, a controversial debate on the actual C sink strength of actively managed boreal forests has arisen (Bellassen & Luyssaert, 2014), fuelled particularly within Sweden by recent single‐site studies suggesting both clear‐cut and mature boreal forests to act as significant C sources (Hadden & Grelle, 2016, 2017; Lindroth et al., 2020; Vestin et al., 2020) and a model simulation proposing that greatest short‐term climate benefits are achieved by reduced harvest levels in productive forests (Skytt et al., 2021). Thus, there is a critical need for reconciling the C balance of actively managed boreal forests to improve our understanding of their potential for mitigating climate change.

A mechanistic understanding of patterns in net ecosystem production (NEP; a proxy for the ecosystem C sink‐source strength) requires detailed knowledge of the dynamics in its underlying component fluxes. These include gross primary production (GPP) and net primary production (NPP) by trees and understory vegetation as well as respiration from plant autotrophic (RA) and microbial heterotrophic (RH) processes (Chapin et al., 2011). The original (Odum, 1969) and extended (Chapin et al., 2011) theories on how these component fluxes regulate the successional changes in forest NEP have been evaluated in several North American chronosequence (Amiro et al., 2010; Bond‐Lamberty et al., 2004; Goulden et al., 2011; Peichl, Arain, & Brodeur, 2010; Peichl, Brodeur, et al., 2010) and global flux synthesis studies (Luyssaert et al., 2007; Xu et al., 2014). These observations have led to contrasting conclusions and show deviations from model theory creating uncertainty specifically on the C source‐sink strength of recently harvested (i.e., clear‐cut) and old (i.e., ≥130 years) forest stands (Coursolle et al., 2012; Curtis & Gough, 2018; Goulden et al., 2011; Luyssaert et al., 2008; Pregitzer & Euskirchen, 2004). In comparison, comprehensive studies testing these concepts in the more actively managed forest landscapes of boreal Fennoscandia are lacking.

Despite the valuable insights into variations of the ecosystem‐scale C balance provided by eddy covariance (EC) studies (Gough et al., 2008; Lindroth et al., 2020; Luyssaert et al., 2007), we still lack detailed data on how this variability is regulated by its separate underlying production and respiration fluxes. Specifically, empirical estimates of RH, that jointly with plant production defines NEP, are relatively limited for boreal forests and instead often assumed as a constant fraction of ecosystem respiration (Amiro et al., 2010), indirectly derived from the difference in EC‐based NEP and biometric NPP estimates (Goulden et al., 2011) or modeled (Luyssaert et al., 2007). Similarly, the contributions from understory production and respiration to the ecosystem C balance may be important in boreal forests (Chi et al., 2021; Nilsson & Wardle, 2005), but are rarely assessed in detail. Thus, there is a need for better knowledge of these individual components of the boreal forest C cycle to improve predictions of whole ecosystem responses to external perturbations.

Another key challenge in C cycle studies is to understand the variability of NEP and regulating drivers at the landscape scale (i.e., tens of km^2^; Goulden et al., 2011). Chronosequence studies are valuable, but often suffer from poor replication and have limited resolution along the successional gradient (Walker et al., 2010). Meanwhile, synthesis studies pooling data across continental and global scales are prone to confounding effects from the various factors related to site characteristics, climate, and management (Magliocca et al., 2015). While most studies conclude that stand age is the dominant control on forest NEP (Besnard et al., 2018; Coursolle et al., 2012), others propose alternative drivers including soil nutrient availability (Fernández‐Martínez et al., 2014; Janssens et al., 2010; Vicca et al., 2012), tree species (Curtis & Gough, 2018), and/or climatic factors (Coursolle et al., 2012). A thorough assessment of how these factors in concert regulate variations of NEP across the managed boreal forest landscape is currently missing.

In this study, we used a comprehensive dataset of biometric‐ and chamber‐based C flux measurements collected in 50 forest stands (ranging from 5 to 211 years) over 3 years (2016–2018) with the aim to explore NEP patterns across a 68 km^2^ managed boreal forest landscape in northern Sweden. The specific objectives were to (1) determine the successional and landscape‐scale variations of NEP; (2) disentangle the main abiotic and biotic drivers regulating NEP at the landscape scale; and (3) estimate the C sequestration potential of boreal rotation‐forestry stands.

## 2 MATERIALS AND METHODS

### 2.1 Study site

**FIGURE 1:**
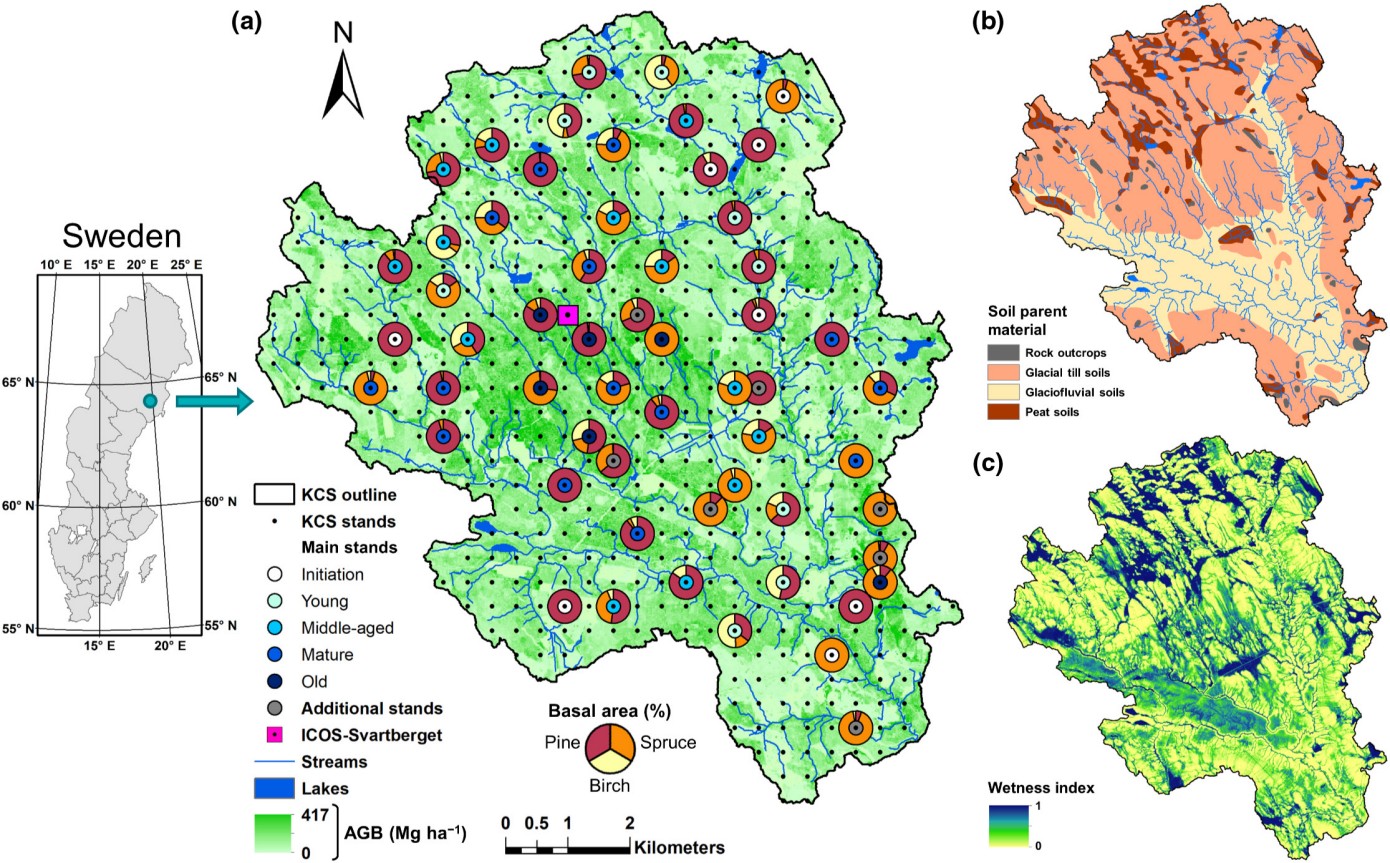
Map of the Krycklan Catchment Study (KCS) located in northern Sweden illustrating (a) the landscape‐variations in aboveground biomass (AGB; data obtained from the Swedish University of Agricultural Sciences and Swedish Forest Agency) and the location, stand age class and species composition of the 50 selected main forest stands, (b) distribution of the predominant soil types (i.e., soil parent material; data obtained from the Swedish Geological Survey), and (c) variations in the soil wetness index (http://www.slu.se/mfk; Ågren et al., 2021). In addition, the seven additional old stands (Section S2), the ICOS‐Svartberget (Integrated Carbon Observation System) Atmosphere‐Ecosystem station, streams, and lakes within the KCS are also shown.

The study was carried out in the Krycklan Catchment Study (KCS, see Table 1 for a list of acronyms) area located in boreal Sweden (64°14′ N, 19°46′ E; Figure 1a), which encompasses a typical actively managed forest landscape in this region (Laudon et al., 2013, 2021). The climate is cold temperate humid with a 30‐year (1991–2020) mean annual precipitation of 638 ± 40 mm and a mean annual air temperature of 2.4 ± 0.3°C. The terrain is gently undulating and spans elevations from 138 m.a.s.l. at the catchment outlet in the southeastern part of the KCS to 339 m.a.s.l. in the northwest. Till‐soils (58% of the total catchment area) dominate the higher‐elevation areas whereas sediment soils (30%) occur mainly in the lower valley regions (Figure 1b). The landscape is dominated by a mosaic of forests (87%) with contributions from mires (9%), lakes (1%), agricultural lands (2%), and rock outcrops (1%). The forest stands include predominantly Scots pine (*Pinus sylvestris* L., 63%) and Norway spruce (*Picea abies* (L.) H. Karst., 26%) mixed with deciduous trees (*Betula* spp., *Alnus incana* (L.) Moench., and *Populus tremula* L., 11%). The understory layer consists mainly of ericaceous shrubs such as bilberry (*Vaccinium myrtillus* L.) and lingonberry (*Vaccinium vitis‐idaea* L.) on moss‐mats of *Hylocomium splendens* (Hedw.) Br. Eur. and *Pleurozium schreberi* (Brid.) Mitt. Most of the forested area is managed by conventional rotation forestry including predominantly even‐aged stands which are mostly artificially regenerated and thinned. Clear‐cutting was carried out as the standard harvest method during which all trees were cut and removed except for a few individual retention trees. The mires are commonly oligotrophic minerogenic and dominated by *Sphagnum* species and a sparse cover of sedges and dwarf shrubs. A network of small streams, both naturally‐formed and man‐made ditches, connect the 13 partially nested sub‐catchments (ranging from 0.1 to 19.1 km^2^) to the river network and regulate landscape hydrology and soil wetness (Figure 1c; http://www.slu.se/mfk Ågren et al., 2014).

**TABLE 1:** Overview of acronyms, definitions, and units for the general terms, carbon fluxes, potential abiotic and biotic drivers, and other variables measured in this study.

Acronym	Definition	Units
*General terms*		
C	Carbon	–
CO_2_	Carbon dioxide	–
KCS	Krycklan Catchment Study	–
I	Initiation stands	–
Y	Young stands	–
Ma	Middle‐aged stands	–
M	Mature stands	–
O	Old stands	–
*Carbon fluxes*		
NEP	Net ecosystem production	g C m^−2^ year^−1^
NPP	Net primary production	g C m^−2^ year^−1^
NPP_t_	Net primary production of trees	g C m^−2^ year^−1^
NPP_u_	Net primary production of understory	g C m^−2^ year^−1^
ANPP_t_	Aboveground net primary production of trees	g C m^−2^ year^−1^
L	Litterfall	g C m^−2^ year^−1^
ANPP_u_	Aboveground net primary production of understory	g C m^−2^ year^−1^
BNPP_t‐cr_	Belowground net primary production of coarse roots of trees	g C m^−2^ year^−1^
BNPP	Belowground net primary production of fine roots (≤2 mm diameter)	g C m^−2^ year^−1^
BNPP_t‐fr_	Belowground net primary production of fine roots of trees	g C m^−2^ year^−1^
BNPP_u_	Belowground net primary production of understory	g C m^−2^ year^−1^
RH	Total heterotrophic respiration	g C m^−2^ year^−1^
RH_s_	Heterotrophic soil respiration	g C m^−2^ year^−1^
RH_dw_	Heterotrophic dead wood respiration	g C m^−2^ year^−1^
*Potential abiotic and biotic drivers*		
Slope	Terrain slope	degrees
N–S	Cosine of the aspect (i.e., north–south gradient)	+1 to −1
E–W	Sine of the aspect (i.e., east–west gradient)	+1 to −1
B_t_	Total tree biomass stock	Mg ha^−1^
LAI_max_	Leaf area index at peak growing season	m^2^ m^−2^
O_depth_	Depth of the organic soil layer	cm
C:N	Average soil carbon‐nitrogen ratio up to 20 cm depth	–
BD	Average soil bulk density up to 20 cm depth	g cm^−3^
*Other variables*		
BA	Stand basal area	m^2^ ha^−1^
Ta_bc_	Below‐canopy air temperature	°C
Ts	Soil temperature at 10 cm depth	°C
SWC	Soil volumetric water content at 5 cm depth	%
SOC	Soil organic carbon up to 20 cm depth	Mg C ha^−1^

We selected 50 forest stands ranging in age (i.e., the number of years after stand establishment) from 5 to 211 years from a regular grid (350 m × 350 m) of 556 permanent forest inventory plots spanning the KCS (Martínez‐García et al., 2022). These stands were grouped into five age classes including initiation (*n* = 8), young (*n* = 9), middle‐aged (*n* = 13), mature (*n* = 14), and old (*n* = 6), which ranged from 5 to 27, 31 to 58, 61 to 78, 80 to 105, and 131 to 211 years old, respectively. Additionally, each stand was classified according to soil type (sediment and till, *n* = 15 and 35, respectively) and dominant tree species (pine and spruce, *n* = 28 and 22, respectively). We note that one initiation‐sediment–pine stand was thinned in spring 2018.

### 2.2 Biometric and chamber‐based estimates of NEP

We determined the annual net ecosystem production (NEP, Equation 1) for each of the 50 forest stands over the 3‐year study period (2016–2018) from the balance between total NPP and total heterotrophic respiration (RH) based on a combination of forest inventory and chamber‐based C flux measurements. In this study, we present both NPP and RH as positive fluxes to facilitate a direct comparison of their magnitudes, whereas positive and negative NEP represent net C uptake and emission, respectively.

(1) NEP=NPP−RH

#### 2.2.1 Net primary production

We estimated annual NPP as the sum of the annual NPP of trees (NPP_t_) and understory vegetation (NPP_u_), (Equation 2).

(2) $$\mathrm{NPP}={\mathrm{NPP}}_{t}+{\mathrm{NPP}}_{u}$$

Specifically, we derived tree above‐ and belowground (coarse root) NPP (ANPP_t_ and BNPP_t‐cr_, respectively) from the annual changes in above‐ and belowground pools of living biomass and dead standing trees (with diameter at breast height, DBH, ≥3 cm) and annual detritus production via litterfall (L). For this purpose, we quantified the annual changes in DBH for all living and dead trees within permanent inventory plots (radius = 10 m) from repeated forest inventories conducted during 2016–2018 in the 47 oldest forest stands (where tree DBH was ≥3 cm) and from tree increment cores (*n* = 150, taken from 2 to 4 representative trees per plot in autumn 2020). We then applied established species‐specific allometric biomass equations for Fennoscandia to obtain whole‐tree biomass estimates (Marklund, 1988; Petersson & Ståhl, 2006; Repola, 2008). We further combined the dry mass and/or volume estimates for dead standing trees with species‐ and decay class‐specific wood densities (Sandström et al., 2007) to estimate above‐ and belowground biomass pools of standing and downed dead trees. Annual L was quantified by using three circular funnel‐shaped litter traps (each with an area of 0.25 m^2^) per inventory plot. In addition, annual tree biomass and L in 3 recent clear‐cuts (5–7 years‐old) were estimated based on the relationships of these components with stand age obtained from the remaining forest stands within the initiation age class. More detailed information on the approach for estimating NPP_t_ is provided in Section S1.

Annual NPP_u_ in the 50 forest stands was derived from changes in the above‐ and belowground biomass pools of understory vegetation as described in detail by Martínez‐Garcia et al. (2022). In brief, we determined the aboveground biomass of all understory plants (i.e., herbs and dwarf shrubs), mosses, and lichens by destructive clipping of subplot quadrats (area 0.25 m^2^; *n* = 3 per inventory plot) in June and August 2017. Biomass production of herbs was assumed to be equal to their peak biomass in August, whereas lichen, moss, and shrub biomass production was calculated from their respective biomass pool changes between June and August. Biomass production of each plant functional type was then summed up to obtain the annual aboveground NPP of understory (ANPP_u_) in 2017. For 2016 and 2018, we estimated aboveground biomass production of dwarf shrubs based on annual shoot length increments conducted in 2018, while aboveground biomass production for the other understory species was obtained by assuming constant ratios between the production of these species and that of dwarf shrubs during the 3‐year study period (see Martínez‐García et al., 2022 for details).

Belowground fine root (≤2 mm diameter) production (BNPP_fr_) of both trees and understory in the 50 forest stands was quantified using the ingrowth core method (Neill, 1992; Vogt et al., 1998). For that purpose, three cores (diameter 10 cm, length 30 cm) were dug and filled with root‐free native soil in each inventory plot in June 2017. These cores were extracted in autumn 2018 to determine the root biomass ingrowth for each core. The ratio of BNPP_fr_ to ANPP (i.e., ANPP_t_ + ANPP_u_) in 2018 was used to derive BNPP_fr_ based on ANPP during 2016–2018. BNPP_fr_ was further partitioned into understory (BNPP_u_) and tree (BNPP_t‐fr_) components. This was achieved by first determining the ratio of above‐ and belowground understory production (i.e., BNPP_u_ and ANPP_u_) in the recent clear‐cut stands. Assuming this ratio to be constant, we then estimated BNPP_u_ based on ANPP_u_ in the remaining stands. BNPP_t‐fr_ was then derived as the difference between BNPP_fr_ and BNPP_u_. Further details for this approach have been described in Martínez‐García et al. (2022).

We then estimated annual NPP_t_ as the sum of ANPP_t_, BNPP_t‐cr_, and BNPP_t‐fr_ (Equation 3), whereas annual NPP_u_ was calculated as the sum of ANPP_u_ and BNPP_u_ (Equation 4).

(3) $${\mathrm{NPP}}_{t}={\text{ANPP}}_{t}+{\text{BNPP}}_{t-\mathrm{cr}}+{\text{BNPP}}_{t-\mathrm{fr}}$$

(4) $${\mathrm{NPP}}_{u}={\text{ANPP}}_{u}+{\text{BNPP}}_{u}$$

The dry mass of standing and downed dead trees were converted to C by using species‐specific C concentrations for each decay class (Mäkinen et al., 2006; Sandström et al., 2007), whereas dry biomass of the remaining NPP components was assumed to have a C concentration of 50%. In our approach, we assumed absent or negligible C losses to herbivory, biogenic volatile organic compounds, and root exudation.

#### 2.2.2 Heterotrophic respiration

We further quantified the total heterotrophic respiration (RH) in the 50 forest stands as the sum of heterotrophic respiration fluxes from soil (RH_s_) and dead wood (RH_dw_) following Equation 5.

(5) $$\mathrm{RH}={\mathrm{RH}}_{s}+{\mathrm{RH}}_{\mathrm{dw}}$$

RH_s_ was estimated every 3–4 weeks during the snow‐free period (early May to late October) based on manual chamber measurements of CO_2_ fluxes from experimental plots (1 m^2^) located few meters outside of the inventory plot (to avoid disturbance to inventory tree roots), where all living vegetation was clipped and roots were trenched along the plot border (Martínez‐García et al., 2022). CO_2_ fluxes were measured with an opaque custom‐made closed steady‐state chamber (45 × 45 × 20 cm) connected to a portable infrared gas analyser (IRGA). Three different IRGAs (MI70, Vaisala; LGR‐GGA‐24EP, Los Gatos Research Inc., GasScouterTM G4301, Picarro) were used over the 3‐year study period. A previous cross‐comparison among the three analysers suggested no significant differences in fluxes (Martínez‐García et al., 2022). During each flux measurement, below‐canopy air temperature (Ta_bc_, °C), soil temperature at 10 cm depth (Ts, °C), and soil volumetric water content at 5 cm depth (SWC, %) were recorded. We then developed plot‐specific Arrhenius response functions (Lloyd & Taylor, 1994) that relate RH_s_ to soil temperature (which was identified as a better explanatory factor than Ta_bc_), to extrapolate RH_s_ based on continuous half‐hourly soil temperature records to annual sums. Details for both the field chamber measurements and model extrapolations were previously described in Martínez‐García et al. (2022). Annual RH_dw_ was derived by multiplying the annual above‐ and belowground dead wood C pools with species‐ and decay class‐specific decomposition rate constants (Shorohova et al., 2008; Yatskov et al., 2003).

#### 2.2.3 Supporting NEP estimates from additional old forest stands

Since our original selection of 50 forest stands only included two old stands with age > 160 years, we selected seven additional forest stands ranging between 169 and 202 years from the KCS' inventory plot network with the aim to reconcile our NEP estimate for the old age class (Figure 1a). For that purpose, we first determined annual ANPP_t_ and BNPP_t‐cr_ in these plots from repeated forest inventory measurements and tree coring in an analogous way to the 50 main forest stands. We then applied fixed ratios and functional relationships (based on stand basal area, BA) defined from the 50 forest stands to derive the annual estimates for remaining NPP_t_ and NPP_u_ components (i.e., BNPP_t‐fr_, L, ANPP_u_, and BNPP_u_) in these seven additional forest stands. Next, the annual RH_dw_ of standing dead trees was estimated with the same approach used for the 50 forest stands. Annual RH_dw_ of downed dead trees and RH_s_ were modeled based on their functional relationships with BA. Finally, annual NEP was calculated from the balance of measured and modelled components of NPP and RH. A detailed description of this approach for estimating NEP at these seven additional old stands is provided in the Section S2.

#### 2.2.4 Ancillary measurements of stand properties

At each soil core location, we further determined soil C and N concentrations as well as bulk density (BD) of the organic and upper mineral (0–10 and 10–20 cm) soil layers as described in detail by Martínez‐García et al. (2022). The depth of the soil organic layer (O_depth_) was also recorded. In August 2017, the leaf area index at peak growing season (LAI_max_) was directly quantified in a subset of 25 forest stands using an LAI‐2200 plant canopy analyser (Li‐Cor Inc.) as described by Aguinaga‐Gil (2018), whereas the remaining 25 forest stands were indirectly estimated from the relationship between LAI_max_ and aboveground tree biomass (see Section S3 for further details). A 0.5 m resolution airborne LiDAR‐derived digital elevation model was used to determine terrain slope and aspect (north–south and east–west gradients).

#### 2.2.5 Statistical analysis

All statistical analyses were based on the 3‐year (2016–2018) mean values. Non‐linear regressions Marquardt method were used to express the mean age‐related changes in NPP, NPP_t_, NPP_u_, and RH_dw_ (Equation 6). For the RH and RH_s_ fluxes, second‐degree polynomial regressions were selected to describe their age‐related trends. The NEP trend line was then computed as the difference between the NPP and RH trend lines.

(6) $$\mathrm{NPP},{\mathrm{NPP}}_{t},{\mathrm{NPP}}_{u},{\mathrm{RH}}_{\mathrm{dw}}=\exp\left(b_{0}+b_{1}\times \ln\left(\mathrm{Age}\right)+b_{2}\times \ln{\left(\mathrm{Age}\right)}^{2}\right)$$

We further conducted primary and secondary principal component analyses (PCAs) with Varimax factor rotation to determine the explanatory factors regulating landscape‐variations in NEP and NPP_t_. The potential abiotic and biotic factors included: terrain slope (slope, degrees), cosine of the aspect (N–S, north–south gradient in +1 to −1 units), sine of the aspect (E–W, east–west gradient in +1 to −1 units), total tree biomass stock (B_t_, Mg ha^−1^), leaf area index at peak growing season (LAI_max_, m^2^ m^−2^), depth of the soil organic layer (O_depth_, cm), average soil carbon‐nitrogen ratio up to 20 cm depth (C:N, dimensionless), and average soil BD up to 20 cm depth (BD, g cm^−3^). In the primary PCA, the NEP components were also included: NPP (g C m^−2^ year^−1^), NPP of trees (NPP_t_, g C m^−2^ year^−1^), NPP of understory (NPP_u_, g C m^−2^ year^−1^), heterotrophic respiration (RH, g C m^−2^ year^−1^), heterotrophic soil respiration (RH_s_, g C m^−2^ year^−1^), and heterotrophic dead wood respiration (RH_dw_, g C m^−2^ year^−1^). In addition, Pearson's correlation analyses were performed between NEP and NPP_t_ and their potential biotic and abiotic factors for all stands and each stand age class. All data analysis was conducted using the Statgraphics Centurion software (version XVI; StatPoint Technologies Inc.).

## 3 RESULTS

### 3.1 Successional changes in NEP, NPP, and RH

**FIGURE 2:**
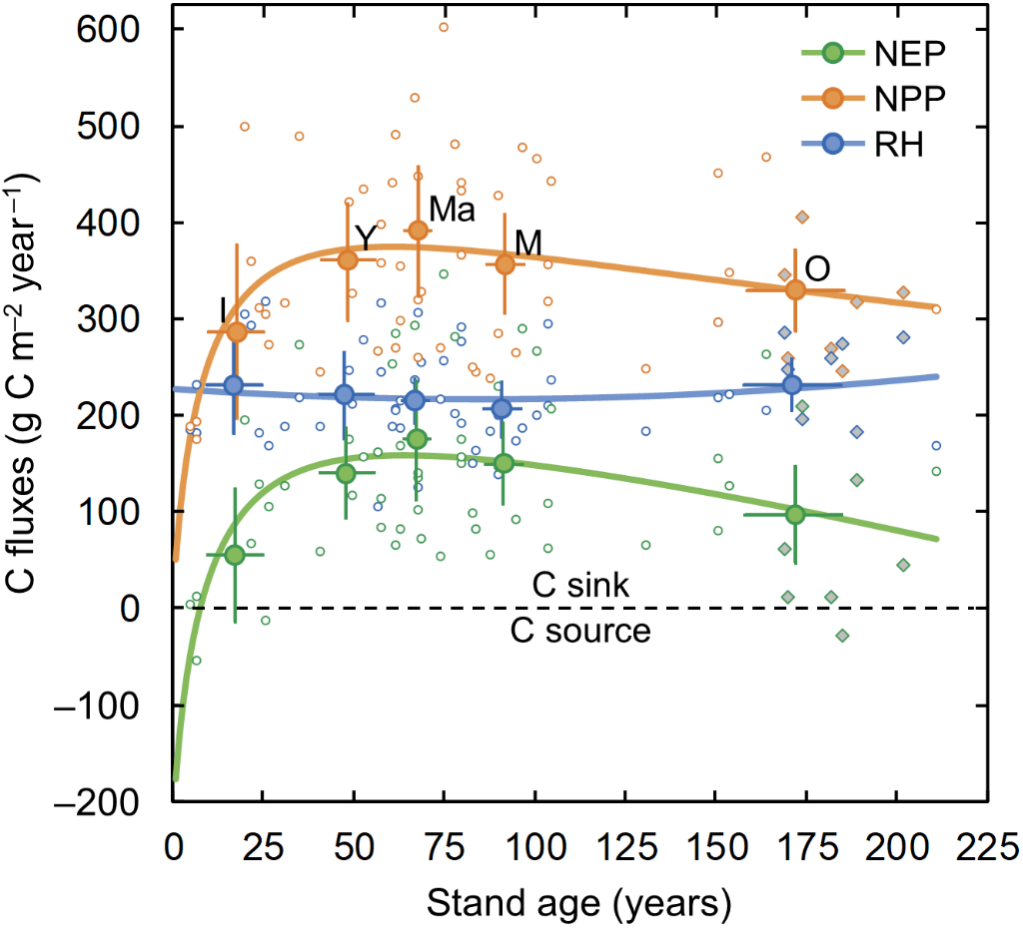
Annual net ecosystem production (NEP) and its components net primary production (NPP) and total heterotrophic respiration (RH) over stand age. Circular open symbols indicate 3‐year means (2016–2018) at each of the 50 main forest stands, circular closed symbols are means for each of the age classes ‘initiation (I)’, ‘young (Y)’, ‘middle‐aged (Ma)’, ‘mature (M)’ and ‘old (O)’. Filled diamonds indicate data for the seven additional old stands (Section S2). Bars represent 95% confidence intervals. Lines for NPP and RH represent non‐linear best fits for the stand‐level data (see supplementary Table S1 for further details). The fit line for NEP was computed as the difference between the lines of NPP and RH.

Our measurements suggest that most of the 50 studied stands had a positive NEP on a 3‐year average, that is, acted as C sinks, except for a recent clear‐cut (7‐year old), a 26‐year‐old stand that was thinned in spring 2018, and one of the seven additional old stands (185‐years old, Figure 2). Overall, the variations of NEP across stand age resembled the classic successional pattern including an initial net C emission period, followed by a sharp increase of NEP during the subsequent few decades, a peak in middle‐age stands (with an age class mean of 174 ± 62 g C m^−2^ year^−1^; mean ± 95% confidence interval) and a gradual decline in the old stands (i.e., the six main and seven additional stands). The initial emission period was limited to less than 10 years and all initiation stands acted on average as a net C sink. Furthermore, NEP in the old stands (*n* = 13) remained at a mean of 98 ± 50 g C m^−2^ year^−1^, equalling 56% of peak NEP in the middle‐aged stands.

Our data further demonstrate that the age‐related changes in NEP corresponded to the dynamics of NPP rather than those of RH (Figure 2). The latter exhibited no significant age‐related trend with mean RH values for the different age classes remaining within a relatively limited range (206 ± 29 to 232 ± 52 g C m^−2^ year^−1^). In comparison, NPP increased rapidly during the first decades, peaked in the middle‐age class (mean age class of 391 ± 68 g C m^−2^ year^−1^), and thereafter gradually declined in mature and old stands, that is, analogue to the patterns of NEP.

**FIGURE 3:**
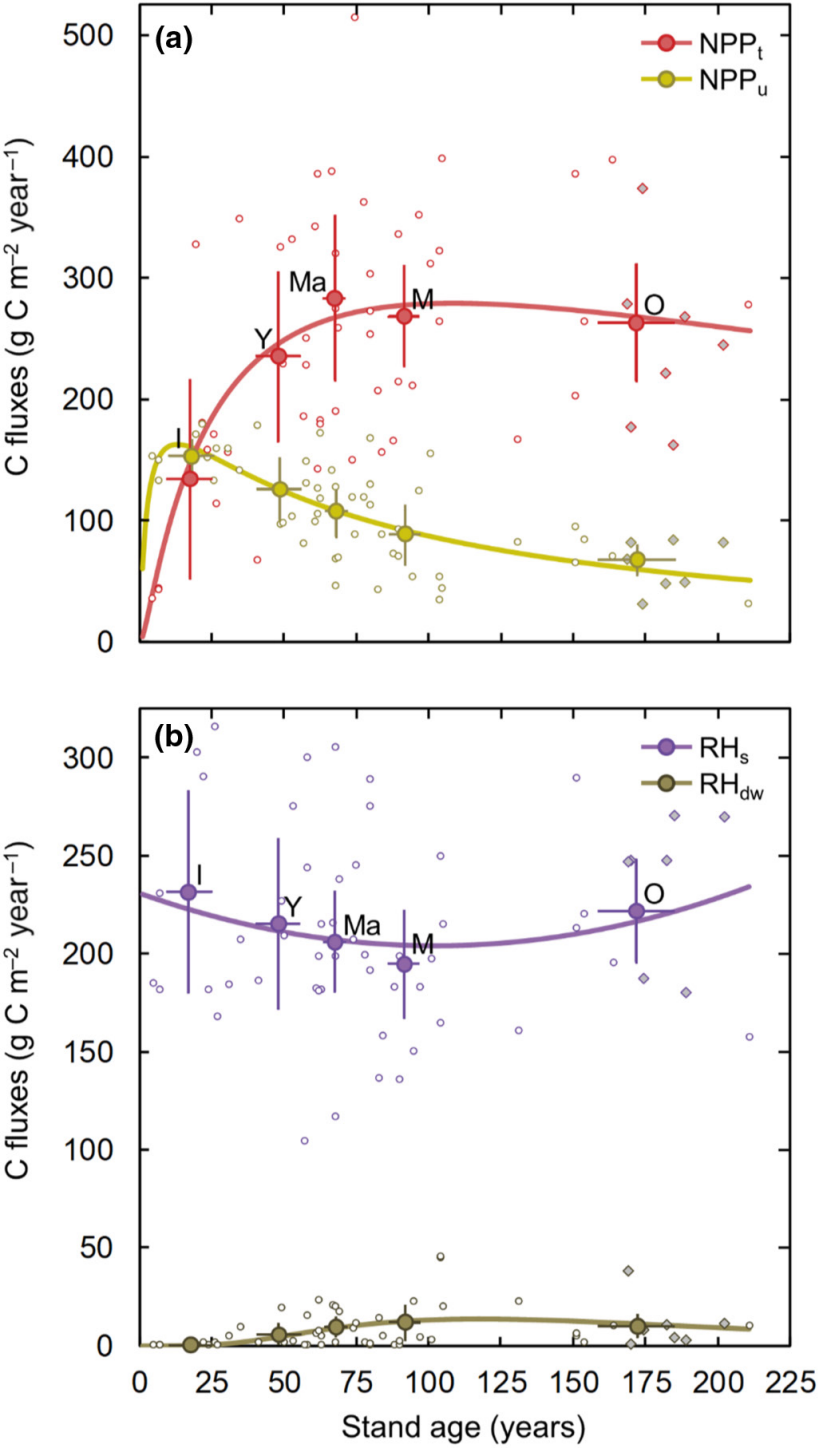
(a) Annual net primary production of trees (NPP_t_) and understory (NPP_u_) and (b) heterotrophic respiration from soil (RH_s_) and dead wood (RH_dw_) over stand age. Circular open symbols indicate 3‐year means (2016–2018) at each of the 50 main forest stands, circular closed symbols show the 3‐year means for each of the age classes ‘initiation (I)’, ‘young (Y)’, ‘middle‐aged (Ma)’, ‘mature (M)’ and ‘old (O)’. Filled diamond symbols indicate the 3‐year mean for the seven additional old stands (Section S2). Bars represent 95% confidence intervals. Lines represent non‐linear best fits for the stand‐level data (see Table S1 for further details).

The further separation of NPP into contributions from tree (NPP_t_) and understory (NPP_u_) production revealed a mirror‐type pattern across stand age (Figure 3a). Specifically, while NPP_u_ exceeded NPP_t_ in the recent clear‐cut stands (and was on average approximately equal for the initiation age class), NPP_u_ and NPP_t_ decreased and increased exponentially, respectively, over stand age. In combination, the divergent patterns in NPP_t_ and NPP_u_ resulted in a relatively limited change in total NPP within the middle‐ to old‐age classes. It is noteworthy that given its relatively greater change, NPP_u_ rather than NPP_t_ forced that gradual small decline of total NPP across these two oldest age classes.

The lack of a clear age‐related change in RH also resulted from opposite trends in its main components (Figure 3b). Specifically, the decreasing trend in soil heterotrophic respiration (RH_s_) between the initiation and mature age classes, followed by an increase in the oldest age class, was counterbalanced by a concurrent increase in RH from dead wood (RH_dw_; including standing and downed dead trees) during the first decades before reaching a somewhat stable level in mature and old stands. Overall, RH_s_ was the dominant component of RH in all age classes whereas the contribution from RH_dw_ was limited to <5%.

### 3.2 Drivers of the landscape‐variability in NEP

**FIGURE 4:**
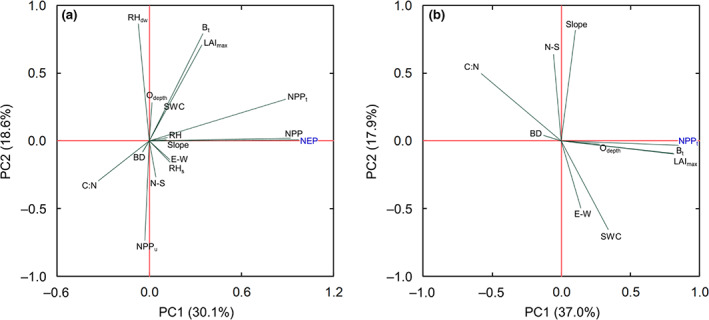
Principal component analysis (PCA) loading plots of controls on (a) forest net ecosystem production (NEP) and (b) tree net primary production (NPP_t_) across the studied managed boreal forest landscape. The input set of potential factors included: Terrain slope (slope, degrees), cosine of the aspect (N–S, north–south gradient in +1 to −1 units), sine of the aspect (E–W, east–west gradient in +1 to −1 units), total tree biomass stock (B_t_, Mg ha^−1^), leaf area index at peak growing season (LAI_max_, m^2^ m^−2^), depth of the soil organic layer (O_depth_, cm), average soil carbon‐nitrogen ratio up to 20 cm depth (C:N, dimensionless), and average soil bulk density up to 20 cm depth (BD, g cm^−3^), net primary production (NPP, g C m^−2^ year^−1^), net primary production of trees (NPP_t_, g C m^−2^ year^−1^), net primary production of understory (NPP_u_, g C m^−2^ year^−1^), total heterotrophic respiration (RH, g C m^−2^ year^−1^), heterotrophic soil respiration (RH_s_, g C m^−2^ year^−1^), and heterotrophic dead wood respiration (RH_dw_, g C m^−2^ year^−1^); *n* = 50 main forest stands

Results from the primary PCA based on the 50 main forest stands suggest that the landscape‐variability of annual NEP was most strongly associated with NPP, and in particular with its NPP_t_ component (Figure 4a). In comparison, the correlations of NEP with RH and NPP_u_ were weak. Furthermore, PCA and supporting Pearson's correlation analysis suggested only a moderate (*r* = 0.35) but significant dependence of NEP on the total tree biomass (B_t_) pool and a lack of significant correlation with the soil C pool (i.e., Odepth; Figure 4a; Table S2). Among the investigated soil properties, the C:N ratio had the strongest effect through a negative relationship with NEP, that is, indicating the highest NEP occurring in the more fertile stands. The significant effects of NPP_t_ and C:N ratio on the landscape‐variability of NEP were further confirmed by Pearson's correlation analysis (Table S2). We did not observe any significant effect of tree species (i.e., pine‐ vs. spruce‐dominated) or soil type (i.e., till vs. sediment soils) on NEP (Figure S1).

Since we revealed NPP_t_ as the main driver of landscape‐scale variations in annual NEP (Figure 4a), we further investigated potential controls of NPP_t_ across the managed landscape. A secondary PCA suggests that B_t_ and leaf area index at peak growing season (LAI_max_) showed the strongest correlations with NPP_t_ across the 50 main forest stands (Figure 4b). Furthermore, a negative correlation between the C:N ratio and NPP_t_ was noted, corresponding to the observed effects of C:N ratio on annual NEP. However, while these three factors explained the landscape‐variability of NPP_t_, the dominant controls of NPP_t_ varied considerably among the different age classes. Specifically, B_t_, LAI_max_, and C:N ratio explained NPP_t_ primarily in initiation, young, and old stands, whereas aspect (i.e., primarily east–west [E–W] and north–south [N–S] gradients) became a more important explanatory factor in middle‐age and mature stands, respectively (Table S3). In initiation stands, soil volumetric water content (SWC, at 5 cm depth) was an additional strong explanatory factor.

### 3.3 Cumulative NEP and C sequestration potential under boreal rotation‐forestry

**FIGURE 5:**
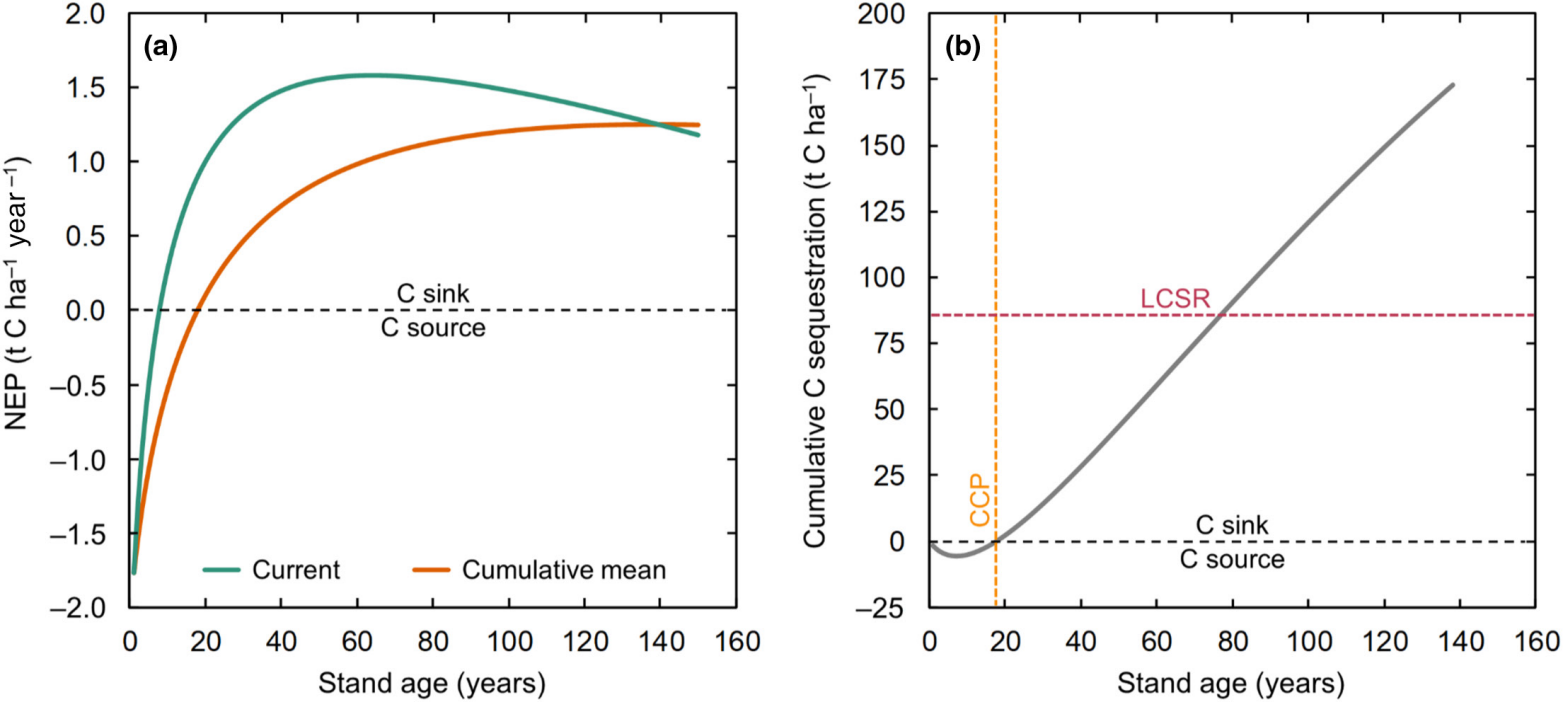
(a) Current and cumulative mean net ecosystem production (NEP, t C ha^−1^ year^−1^) over stand age indicating the period for optimum carbon sequestration until the age of 138 years and (b) cumulative net ecosystem production (NEP, t C ha^−1^) of a boreal forest stand under rotation‐forestry (based on the NEP line (i.e., NPP–RH) from Figure 2) over the optimum rotation period of 138 years. Vertical yellow and horizontal red dotted lines show the C compensation point (CCP, i.e., age at which the cumulative C balance turns from source to sink) and the long‐term C sequestration rate (LCSR, i.e., the mean C accumulation over multiple 138‐year rotation periods).

A comparison of the current annual NEP with the cumulative mean annual NEP trajectories over stand age suggests that the period of optimum C sequestration lasts until the age of 138 years (i.e., at the intersection point of both trajectories), after which the current NEP decreased below the cumulative mean NEP (Figure 5a). The cumulative NEP over such a 138‐year optimum rotation period suggests a total C sequestration of 173 t C ha^−1^ (Figure 5b). Assuming an annual harvest rate of 0.78% (required to maintain a steady state in the tree biomass pool over a 138‐year rotation), the long‐term C sequestration rate (LCSR) was 86.5 t C ha^−1^ per rotation over multiple 138‐year rotation periods. These results further indicate that a period of 18 years is required until the cumulative net C loss from the initial years is compensated by net C uptake in subsequent years, that is, at the C compensation point (CCP), followed by continuous net C gain until the end of the rotation period (Figure 5b).

## 4 DISCUSSION

Despite its important role in climate change mitigation strategies, the C cycle of actively managed boreal forest landscapes, in particular its successional change and net C balance, is currently uncertain and therefore under intense debate. Our results based on detailed C flux measurements in 50 forest stands fill these knowledge gaps by providing empirical evidence for (1) a divergence in the early and late successional NEP dynamics in actively managed relative to naturally regenerating boreal forests; (2) the important roles of both tree and understory production, rather than heterotrophic C emissions, for regulating NEP at the landscape‐scale; and (3) the C sink‐strength of boreal rotation‐forestry stands. Altogether, our findings, therefore, point toward a considerable potential in active forest management of boreal forest landscapes for maximizing C sequestration and associated climate benefits.

### 4.1 Successional changes in the C balance of actively managed boreal forests

A distinct feature of rotation‐forestry is the combination of strong structural gradients in stand age and biomass across the forest landscape and the removal of biomass via harvest. This has consequences for both the uptake and emissions of C via organic matter production and decomposition, respectively, which jointly regulate the forest net C balance. The results from our flux measurements in 50 rotation‐forestry stands (complemented with semi‐empirical estimates for seven additional old stands) illustrate that the successional NEP pattern followed the expected unimodal curve, which agrees with both classic theory (Chapin et al., 2011; Odum, 1969) and observations from other boreal forest regions (Coursolle et al., 2012; Goulden et al., 2011). Furthermore, the observed minimum‐maximum range in NEP (−56 to +346 g C m^−2^ year^−1^) compares well with the 25%–75% range (−61 to +270 g C m^−2^ year^−1^) for 14 boreal humid stands (72 ± 52 years‐old, [mean ± standard deviation], including managed and unmanaged stands) (Luyssaert et al., 2007) and with the upper boundary estimates (−386 to +323 g C m^−2^ year^−1^) from a Scots pine chronosequence (spanning 4–75 years) in hemiboreal Finland (Kolari et al., 2004). Our observation that the patterns in NPP rather than those in RH drive the successional changes in NEP is also in line with the findings from studies in the North American boreal forests regenerating after fire disturbance (Amiro et al., 2010; Goulden et al., 2011) and after clear‐cutting of hemiboreal forests in Estonia (Uri et al., 2022). This suggests that the general trajectory and successional ranges in NEP are comparable between forests with contrasting regeneration and management regimes across the boreal biome. Nevertheless, NEP magnitudes observed in our youngest and oldest age classes deviated from those reported for naturally disturbed or unmanaged boreal forests, which indicates divergent features in the C cycle of actively managed and unmanaged boreal forest landscapes.

Specifically, we noted a positive NEP (i.e., a C sink) in all stands with ages of 5–20 years (except for one 7‐year‐old stand), which contrasts the common perception that boreal clear‐cut areas may act as major C sources for more than 10 years, a notion that is derived primarily from observations in boreal stands regenerating after natural disturbances (Amiro et al., 2010; Coursolle et al., 2012; Goulden et al., 2011; Harmon et al., 2011) and from recent clear‐cuts in temperate regions (Pregitzer & Euskirchen, 2004). One reason for the early switch from source to sink in our rotation‐forestry stands was the rapid establishment of the understory layer, whose productivity rates equaled those of the tree layer during the first two decades. Similar findings on the importance of forest floor vegetation in restoring the C sink following harvest have been noted at hemiboreal sites (Uri et al., 2022). Furthermore, the Swedish Forestry Act requests the planting of new tree seedlings within 3 years after clear‐cutting, which subsequently results in a faster tree layer development compared with other boreal regions subject to less intense forestry (Högberg et al., 2021).

Another reason for the limited emissions in the clear‐cut stands was the absence of enhanced RH. This result contrasts earlier studies reporting increased RH after stand replacing fire disturbance (Harmon et al., 2011) and after harvest in temperate regions (Pregitzer & Euskirchen, 2004). Thus, our study challenges the common perception that large C emissions are a common feature of boreal clear‐cut areas. Instead, a relatively colder climate and nutrient‐poor soils might suppress a strong increase in soil decomposition rates following clear‐cutting in the boreal landscape. In agreement with our findings, relatively stable RH rates were also observed in a nutrient‐limited (C:N ratio = 29) Scots pine chronosquence in hemiboreal Estonia (Uri et al., 2022) and in a synthesis study of RH in the boreal region (Pregitzer & Euskirchen, 2004). It is noteworthy, however, that our study lacks NEP data from the first few years after harvest during which considerably larger emissions have been reported by other studies conducted in boreal (Tong, Nilsson, et al., 2022) and hemiboreal (Tong, Nilsson, Sikström, et al., 2022; Vestin et al., 2020) clear‐cut sites.

The second divergence in our results on successional NEP changes concerns the C sink‐strength of old boreal forests which has been under intense debate and remained highly uncertain (Gundersen et al., 2021; Luyssaert et al., 2008). Specifically, while classic theory and some empirical studies suggest that annual NEP decreases beyond the maturity stage toward close‐to‐zero or even negative balances (Chapin et al., 2011; Hadden & Grelle, 2017; Odum, 1969), other studies report that old stands continue to act as large C sinks (Luyssaert et al., 2008). In support of this discussion, our results demonstrate a continuous net ecosystem C uptake in the oldest age class of our actively managed boreal forests at relatively high rates (~100 g C m^−2^ year^−1^) exceeding those reported from less intensive or unmanaged boreal forests (Coursolle et al., 2012; Goulden et al., 2011). We show that this sustained NEP is primarily explained by the stable mean tree NPP during the ages of 100–200 years. The latter is well‐supported by silvicultural studies (e.g., Stokland, 2021) and the Swedish National Forest Inventory (NFI) data (Figure S2) suggesting that tree volume production rates in boreal rotation‐forestry stands do not decline markedly during the second century of tree growth. The lack of a strong decline in tree NPP in the studied old stands could also be facilitated by relatively low tree mortality rates (Figure S3). An additional reason for sustained NEP might be that the limited build‐up of dead wood in actively managed relative to unmanaged forest stands (Fridman & Walheim, 2000; Jonsson et al., 2016) constrains the increase in RH (and thus reduction in NEP) at the old stand age class. Instead, we note that the modest decrease in NEP in our oldest age class was primarily the result of the diminishing contribution from understory NPP, rather than a reduction in tree NPP or increased RH. This further underlines the important role of the understory dynamics in regulating successional changes in NEP across managed boreal forests. At the biome scale, considerably lower disturbance rates (e.g., fires, wind‐throw, and insect outbreaks) in managed compared with unmanaged old forests (Högberg et al., 2021) may further help to maintain high tree production rates and lower C emissions from smaller debris pools and thus support a greater C sink in managed old boreal stands.

### 4.2 Drivers of the landscape‐variability in the C balance of boreal forests

Considerable knowledge of the stand and mesoscale variations in the boreal forest C balance due to gradients in stand age, climate, and geographical settings has been gathered from chronosequence and global synthesis studies (Coursolle et al., 2012; Goulden et al., 2011; Pregitzer & Euskirchen, 2004; Xu et al., 2014). In comparison, a detailed understanding of the variations in NEP across the heterogeneous landscape is lacking. Our study demonstrates that the observed range of the landscape‐variability (402 g C m^−2^ year^−1^) in annual NEP within our 68 km^2^ boreal forest catchment was similar to the range (~330 g C m^−2^ year^−1^) observed in previous synthesis studies pooling data across various geographical regions within the boreal biome (Luyssaert et al., 2007). This suggests that forest NEP variations in the boreal biome are primarily controlled by intrinsic landscape characteristics relative to geographical and/or climatic gradients. In support, synthesis studies commonly report a lack of strong climatic controls on boreal forest NEP (Luyssaert et al., 2007). Thus, disentangling the drivers of landscape‐variability from those operating at the stand‐ and mesoscale resembles a critical issue for better understanding which factors regulate the net C sink‐strength of a heterogeneous forest landscape.

Our primary PCA results suggest that the landscape‐variability of NEP was most strongly associated with tree NPP. We observed a particularly large variability in NEP at the middle‐age and mature age classes, which demonstrates that divergent tree growth rates have considerable consequences for the forest C balance. This further highlights a considerable potential of optimizing tree biomass production for enhancing C sequestration benefits. Within a forest landscape, tree growth is naturally impeded locally by sub‐optimum conditions related to soil water supply (i.e., too dry or too wet), nutrient availability (e.g., discharge and recharge areas across slopes), and topography (e.g., cold air lakes, north‐facing slopes) (Aussenac, 2000; Hägglund & Lundmark, 1977; Högberg et al., 2017). Our secondary PCA analysis showed that out of a suit of potential factors, tree NPP was regulated mainly by maximum leaf area index and soil nutrient level (i.e., C:N ratio) at the landscape scale. Thus, strategies to enhance boreal forest C sequestration should aim to optimise the landscape‐variability in these key factors and develop appropriate management actions (i.e., tree species selection, thinning, and/or fertilization) that shift the mean NEP‐age curve toward the optimum upper boundary.

In comparison with NPP, the landscape‐scale variation in the C emissions via RH was most limited and not responding strongly to any of the investigated biotic and abiotic factors, despite the presence of relatively large gradients in, for example, soil organic carbon (SOC), O_depth_, litterfall, C:N ratio, Ts, and SWC (Figure S4). This finding appears to be in contrast to numerous stand‐scale and synthesis studies that reported soil temperature and moisture as major drivers of soil RH (Davidson & Janssens, 2006; Harmon et al., 2011). Most often, however, these relationships of RH with abiotic factors primarily explain the temporal (i.e., seasonal) dynamics in RH and are subsequently assumed to hold also at the spatial landscape scale. Our results challenge this assumption. One explanation for the lack of key drivers for the landscape variability in RH in our study could be counteracting effects from various factors. Along the stand age gradient, for instance, the potential for greater RH due to the increasing supply of organic matter (Figure S4a–c) might be counterbalanced by lower decomposition rates because of decreasing litter quality and soil temperature (Figure S4d,e), the latter resulting from increasing canopy shading. This is further supported by our observation that various environmental factors correlate well with RH only within a given age class, but with the dominant factor varying among age classes (Table S4). Altogether, this suggests that, at the spatial scale of an actively managed forest landscape, RH resembles a relatively stable source of C rather than a dynamic component regulating the landscape C balance.

### 4.3 The C sink‐strength of boreal rotation‐forestry

Our data suggest that the studied boreal landscape under conventional rotation‐forestry provided a quasi‐continuous sink for atmospheric CO_2_. Despite the presence of transient local C sources due to clear‐cutting, the initial period of net C loss occurring at the individual stand level following harvest is well overruled by the substantial C uptake by the neighboring older stands at the landscape scale. These findings support a recent synthesis report based on NFI data from Sweden, Finland, Canada, and Russia, which concluded that C sequestration occurs in both soil and biomass at higher rates in actively managed forests compared with low‐intensive and unmanaged forests in the boreal region (Högberg et al., 2021). Our study also agrees well with previous estimates based on tall tower EC flux measurements which suggested that the studied landscape acted as an annual CO_2_ sink of 37 to 122 g C m^−2^ year^−1^ during 2016–2018 (Chi et al., 2019, 2020). Furthermore, our results reconcile with regional scale estimates from dynamic vegetation models constrained by atmospheric CO_2_ concentration measurements, which indicate that the boreal region of Sweden acted as a C sink during the same study years (Sathyanadh et al., 2021). While additional data are urgently needed to support conclusions on the C sink‐strength of actively managed boreal forest, our comprehensive empirical data provide an important first baseline for assessing the climate mitigation potential of boreal rotation‐forestry. It further serves as a valuable reference for evaluating the C sequestration potential of alternative forest management strategies, for example, continuous cover forestry, which are currently under intensive debate within the Fennoscandian region with the goal to enhance other forest ecosystem services, such as biodiversity and societal values.

Our study further revealed that tree NPP was the dominant control of NEP which implies that intensive forestry that enhances tree biomass production will likely outcompete alternative strategies that result in lower tree growth rates. This is in line with previous synthesis studies arriving at similar conclusions when comparing the C sequestration potential of managed and unmanaged forests in other regions (e.g., Xu et al., 2014). In addition, the continued C uptake in older stands observed in our study, combined with the relatively low risk of stand‐replacing natural disturbances in the boreal regions of Fennoscandia (Högberg et al., 2021), indicates that longer rotation periods, that is, exceeding the current soil fertility dependent range of ~100–120 years for boreal rotation‐forestry (Lämås et al., 2015), would be beneficial from the perspectives of C sequestration and climate change mitigation. Overall, our study highlights the key role of forest management in maximizing C sequestration in boreal forests and associated climate mitigation benefits through strategies that optimize tree growth.

## AUTHOR CONTRIBUTIONS

Matthias Peichl, Mats B. Nilsson, and Hjalmar Laudon designed the study. Matthias Peichl provided funding acquisition, project administration, and resources. Tomas Lundmark contributed with additional funding. Matthias Peichl, Mats B. Nilsson, Eduardo Martínez‐García, Johan E.S. Fransson, and Jörgen Wallerman planned and conducted the data collection. Eduardo Martínez‐García and Jörgen Wallerman processed the data. Eduardo Martínez‐García, Matthias Peichl, and Mats B. Nilsson analysed and interpreted the data. Matthias Peichl wrote the manuscript with contributions from all co‐authors.

## CONFLICT OF INTEREST

The authors declare that they have no known competing financial interests or personal relationships that could have appeared to influence the work reported in this paper.

## Supporting information


Appendix S1.

## Data Availability

The data that support the findings of this study are openly available in the Zenodo digital repository at https://doi.org/10.5281/zenodo.7312986. This manuscript does not include any code.
