# Supplementary material for: Landscape‐variability of the carbon balance across managed boreal forests
Source: Glob Chang Biol. 2022 Dec 4;29(4):1119–32. doi: 10.1111/gcb.16534 (PMC10108254; doi:10.1111/gcb.16534)
Supplement: Supplementary file 1 — Appendix S1. [file GCB-29-1119-s001.docx]

Supplementary materials for

**Landscape-variability of the carbon balance across managed boreal forests**

Matthias Peichl^1,*^, Eduardo Martínez-García^1^, Johan E.S. Fransson^2,3^, Jörgen Wallerman^2^, Hjalmar Laudon^1^, Tomas Lundmark^1^, Mats B. Nilsson^1^

^1^ Department of Forest Ecology and Management, Swedish University of Agricultural Sciences, Skogsmarksgränd 17, SE-901 83, Umeå, Sweden

^2^ Department of Forest Resource Management, Swedish University of Agricultural Sciences, Skogsmarksgränd 17, SE-901 83, Umeå, Sweden

^3^ Department of Forestry and Wood Technology, Linnaeus University, Georg Lückligs väg 1, SE-351 95, Växjö, Sweden;

Matthias Peichl (MP); Matthias.Peichl@slu.se; ORCID-ID 0000-0002-9940-5846; ^*^Corresponding author

Eduardo Martínez-García (EMG); Eduardo.Martinez@slu.se; ORCID-ID 0000-0003-2176-9671

Johan E.S. Fransson (JESF); Johan.Fransson@slu.se; ORCID-ID 0000-0002-7913-8592

Jörgen Wallerman (JW); Jorgen.Wallerman@slu.se; ORCID-ID 0000-0002-9996-1447

Hjalmar Laudon (HL); Hjalmar.Laudon@slu.se; ORCID-ID 0000-0001-6058-1466

Tomas Lundmark (TL); Tomas.Lundmark@slu.se; ORCID-ID 0000-0003-2271-3469

Mats B. Nilsson (MBN); Mats.B.Nilsson@slu.se; ORCID-ID 0000-0003-3765-6399

Contents:

Supplementary Figures S1–S4

Supplementary Tables S1–S3

Supplementary methods (Sections 1–3 and Supplementary Tables S4–S13)

References

**Supplementary figures**

***Figure S1.* Box plots representing the effect of a) soil type, b) tree species, c) and stand age on the net ecosystem production (NEP).** Data based on 3-year mean annual values (2016-2018). The boxes represent the 25^th^ (bottom) and 75^th^ (top) percentiles, the central line the median, and the cross the mean. Whiskers below and above the box denote data within 1.5 times of the interquartile range and outliers are given as individual points. Significant effects are highlighted in red. Different superscript letters denote significant differences (Bonferroni test, *p* < 0.05) for each main effect. Horizontal dashed line indicates the carbon (C) source-to-sink transition. *n* = 57 forest stands (i.e., 50 main + 7 additional).


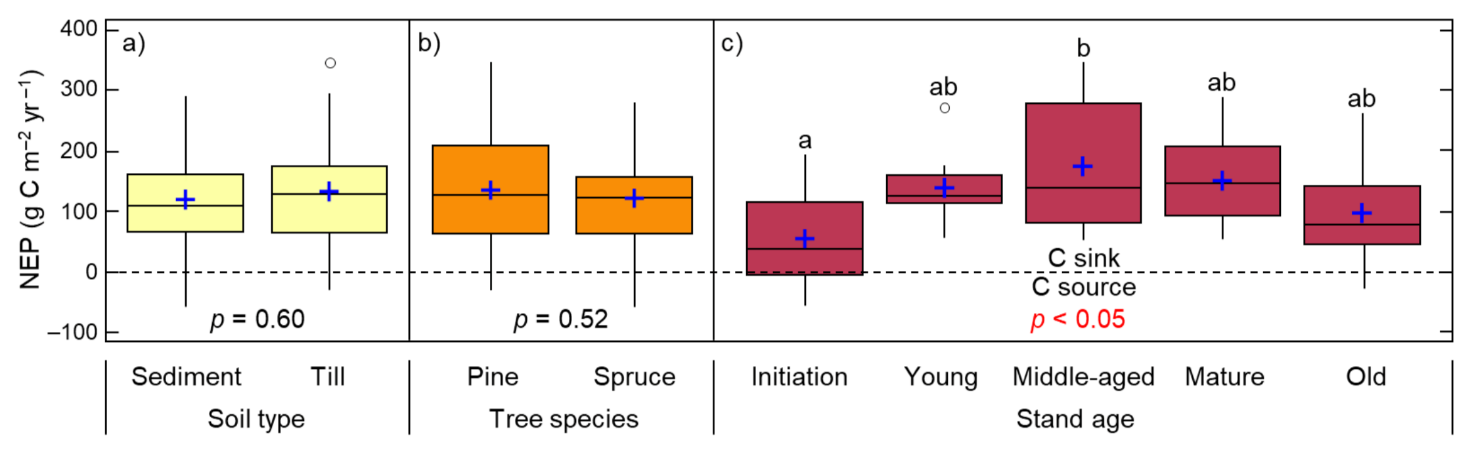


***Figure S2.* Mean annual stem volume increment of forest stands located in northern Sweden in 20–year age classes.** Values (m^3^ ha^–1^ yr^–1^) for the Norrland region and its Northern and Southern parts are shown. Data are based on statistics from productive forest land outside formally protected areas and excluding growth of felled trees. Data for the average year 2015 were used, representing years of increment 2011–2019. Source: Sweden’s National Forest Inventory, 2016-2020 data (https://skogsstatistik.slu.se/pxweb/en/OffStat/).


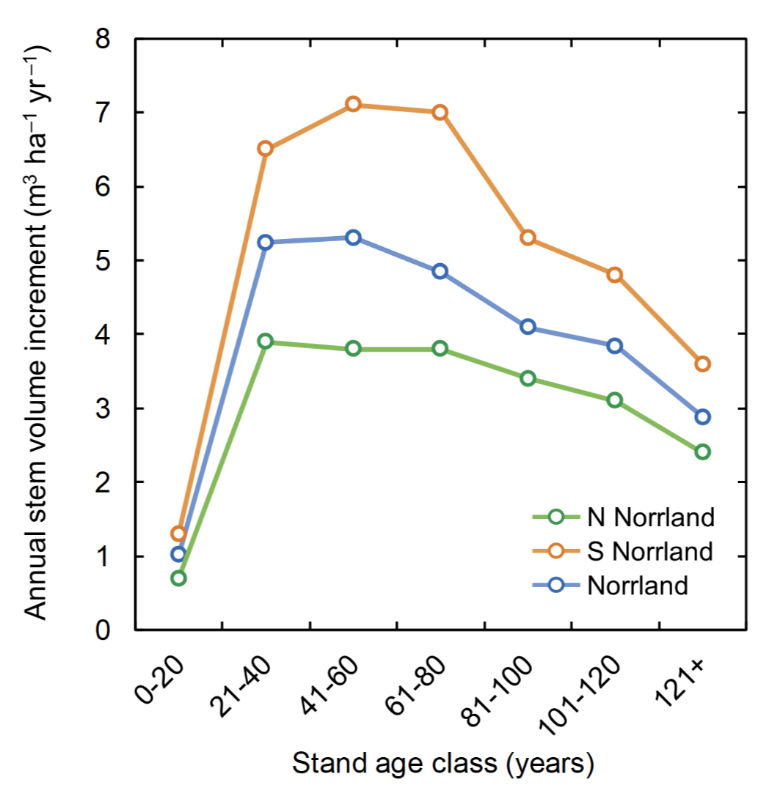


***Figure S3.* Relationship between tree mortality (TM) with stand age.** TM is expressed in terms of loss of basal area per year (% yr^–1^). TM in recent clear-cuts was assumed negligible. Data based on 3-year means (2016–2018). Round open symbols indicate data for the main 50 forest stands, filled diamonds indicate data for the 7 additional old stands (see Supplementary materials, Section 2), whereas round closed symbols show means for each of the stand age classes ‘initiation (I)’, ‘young (Y)’, ‘middle-aged (Ma)’, ‘mature (M)’, and ‘old (O)’. Bars represent 95% confidence intervals. Lines and shaded areas represent best regression fits and confidence intervals for the stand-level data. *n* = 57 forest stands (i.e., 50 main + 7 additional).


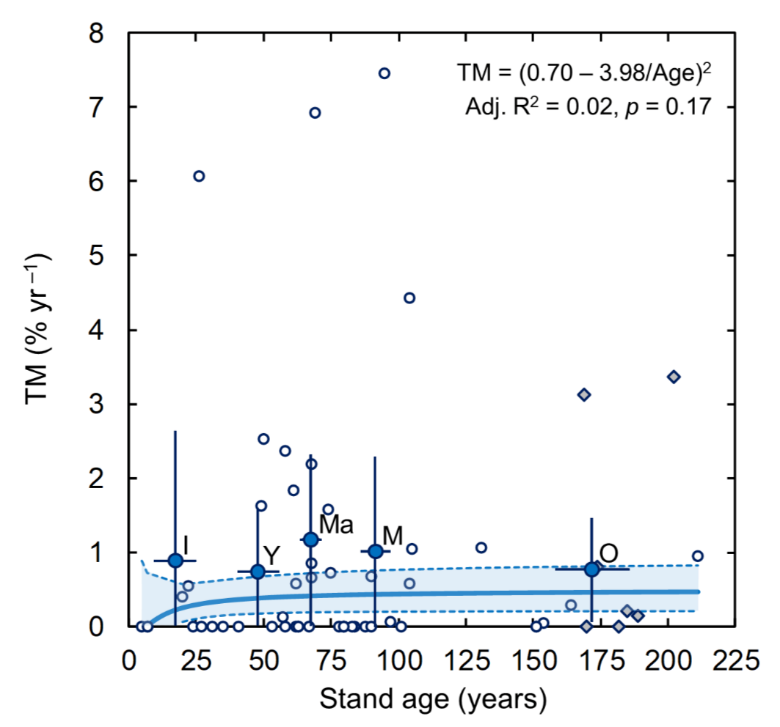


***Figure S4.* Relationships between different abiotic and biotic factors with stand age.** a) soil organic carbon up to 20 cm depth (SOC), b) depth of the soil organic layer (O_depth_), c) annual detritus production via litterfall (litterfall), d) average soil carbon-nitrogen ratio up to 20 cm depth (C:N), e) average annual soil temperature at 10 cm depth (Ts), and f) average annual soil volumetric water content at 5 cm depth (SWC). Round closed symbols indicate means for each of the stand age classes ‘initiation (I)’, ‘young (Y)’, ‘middle-aged (Ma)’, ‘mature (M)’, and ‘old (O)’. Bars represent 95% confidence intervals. Lines and shaded areas represent best regression fits and confidence intervals for the stand-level data. *n* = 50 main forest stands.


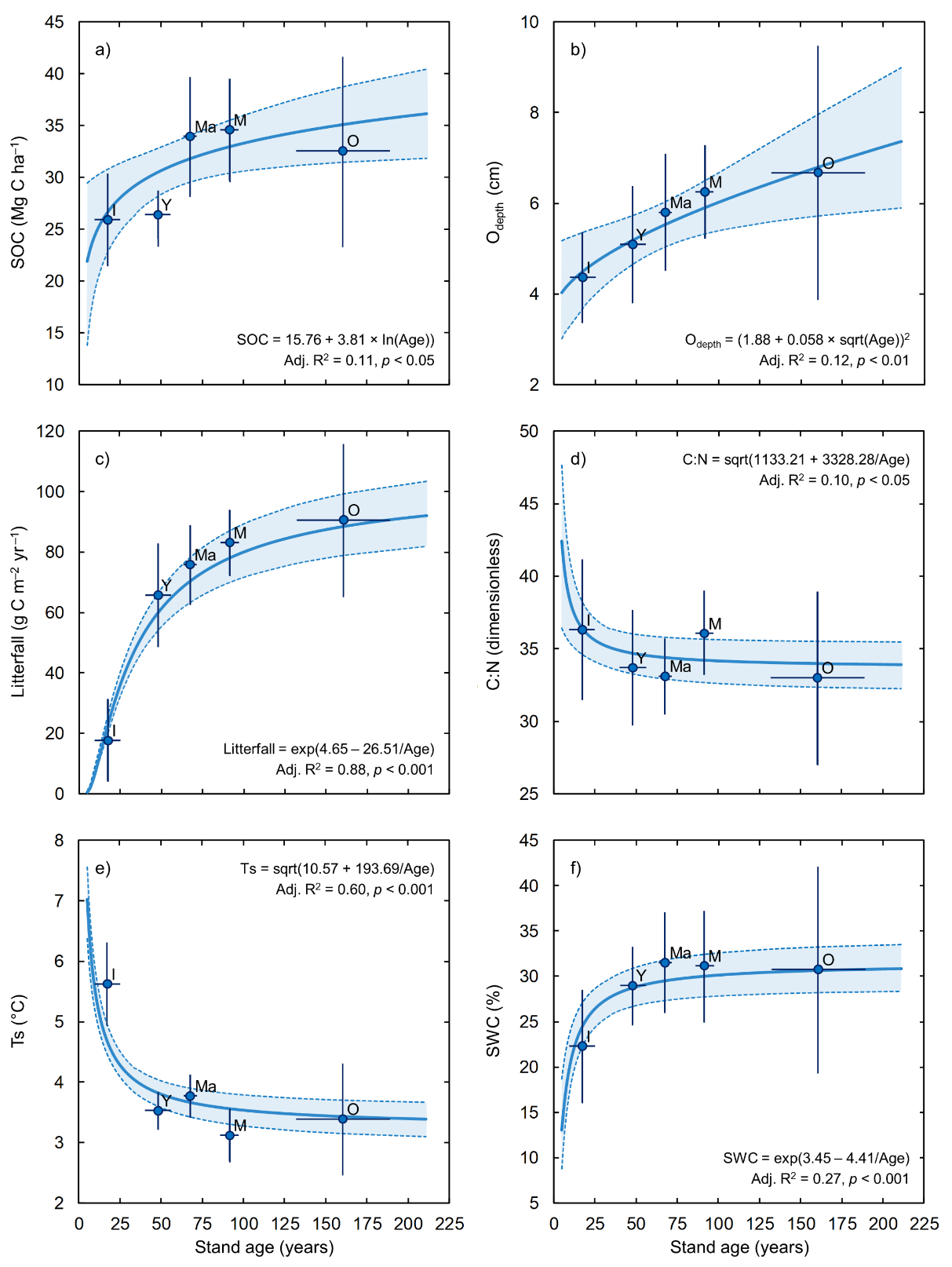


**Supplementary tables**

***Table S1.* Coefficients (b_0_, b_1_, b_2_, b_3_) and their standard errors (SE),** **adjusted coefficients of determination (Adj. R^2^), standard error of estimate (SEE), and *p*-value of linear regressions determined among annual C fluxes (g C m^−2^ yr^−1^) and stand age (Age, years).** Linear regressions are presented in Figs. 2 and 3 in the manuscript. Equation forms are also shown. SE of non-linear regressions (i.e., 1, 3, 4, and 6) are asymptotic standard errors. Non-linear and non-significant regressions are denoted as *nlr* and *ns*, respectively. *n* = 57 forest stands (i.e., 50 main + 7 additional).

|  | **Coefficients** | | | |  | **SE Coefficients** | | | |  | **Adj. R^2^** | **SEE** | ***p*** |
| --- | --- | --- | --- | --- | --- | --- | --- | --- | --- | --- | --- | --- | --- |
| **Regression** | **b_0_** | **b_1_** | **b_2_** | **b_3_** |  | **b_0_** | **b_1_** | **b_2_** | **b_3_** |  |  |  |  |
| 1 | **NPP = exp(b_0_ + b_1_ × ln(Age) + b_2_ × ln(Age)^2^)** | | | | | | | | | | | | |
| 1 | 3.91 | 0.98 | –0.12 | – |  | 0.72 | 0.37 | 0.05 | – |  | 0.15 | 88.6 | *nlr* |
|  |  |  |  |  |  |  |  |  |  |  |  |  |  |
| 2 | **RH = b_0_ + b_1_×Age + b_2_×Age^2^** | | | | | | | | | | | | |
| 2 | 227.40 | –0.27 | 0.002 | – |  | 21.98 | 0.49 | 0.002 | – |  | < 0.01 | 50.7 | *ns* |
|  |  |  |  |  |  |  |  |  |  |  |  |  |  |
| 3 | **NPP_t_ = exp(b_0_ + b_1_ × ln(Age) + b_2_ × ln(Age)^2^)** | | | | | | | | | | | | |
| 3 | 1.43 | 1.79 | –0.19 | – |  | 1.77 | 0.85 | 0.11 | – |  | 0.26 | 86.3 | *nlr* |
|  |  |  |  |  |  |  |  |  |  |  |  |  |  |
| 4 | **NPP_u_ = exp(b_0_ + b_1_ × ln(Age) + b_2_ × ln(Age)^2^)** | | | | | | | | | | | | |
| 4 | 4.10 | 0.77 | –0.15 | – |  | 0.46 | 0.28 | 0.04 | – |  | 0.49 | 30.0 | *nlr* |
|  |  |  |  |  |  |  |  |  |  |  |  |  |  |
| 5 | **RH_s_ = b_0_ + b_1_×Age + b_2_×Age^2^** | | | | | | | | | | | | |
| 5 | 230.83 | –0.52 | 0.003 | – |  | 21.63 | 0.48 | 0.002 | – |  | < 0.01 | 49.9 | *ns* |
|  |  |  |  |  |  |  |  |  |  |  |  |  |  |
| 6 | **RH_dw_ = exp(b_0_ + b_1_ × ln(Age) + b_2_ × ln(Age)^2^)** | | | | | | | | | | | | |
| 6 | –28.45 | 13.02 | –1.37 | – |  | 47.47 | 29.12 | 7.74 | – |  | 0.10 | 10.2 | *nlr* |

***Table S2.* Heat map based on correlation matrix among biotic and abiotic factors controlling the net ecosystem production (NEP).** Warm colours represent positive correlation and cool colours represent negative correlation based on Pearson’s correlation test. Data pooled for all stands and each of the stand age classes (initiation, young, middle-aged, mature, and old). Significant correlations (*p* < 0.05) are marked in bold. Marginally significant correlations (*p* < 0.1) are marked with *. Slope: terrain slope (degrees), N-S: cosine of the aspect (north-south gradient in +1 to –1 units), E-W: sine of the aspect (east-west gradient in +1 to –1 units), LAI_max_: leaf area index at peak growing season (m^2^ m^−2^), B_t_: total tree biomass stock (Mg ha^−1^), SWC: average annual soil volumetric water content at 5 cm depth (%), O_depth_: depth of the soil organic layer (cm), C:N: average soil carbon-nitrogen ratio up to 20 cm depth (dimensionless), and BD: average soil bulk density up to 20 cm depth (g cm^−3^). *n* = 50 main forest stands.


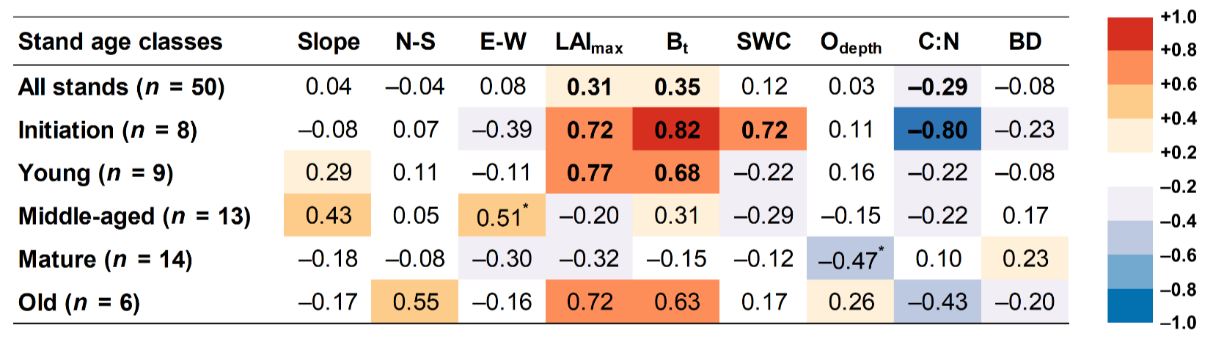


***Table S3.* Heat map based on correlation matrix among biotic and abiotic factors controlling the net primary production of trees (NPP_t_).** Warm colours represent positive correlation and cool colours represent negative correlation based on Pearson’s correlation test. Data pooled for all stands and each of the stand age classes (initiation, young, middle-aged, mature, and old). Significant correlations (*p* < 0.05) are marked in bold. Marginally significant correlations (*p* < 0.1) are marked with *. Slope: terrain slope (degrees), N-S: cosine of the aspect (north-south gradient in +1 to –1 units), E-W: sine of the aspect (east-west gradient in +1 to –1 units), LAI_max_: leaf area index at peak growing season (m^2^ m^−2^), B_t_: total tree biomass stock (Mg ha^−1^), SWC: average annual soil volumetric water content at 5 cm depth (%), O_depth_: depth of the soil organic layer (cm), C:N: average soil carbon-nitrogen ratio up to 20 cm depth (dimensionless), and BD: average soil bulk density up to 20 cm depth (g cm^−3^). *n* = 50 main forest stands.


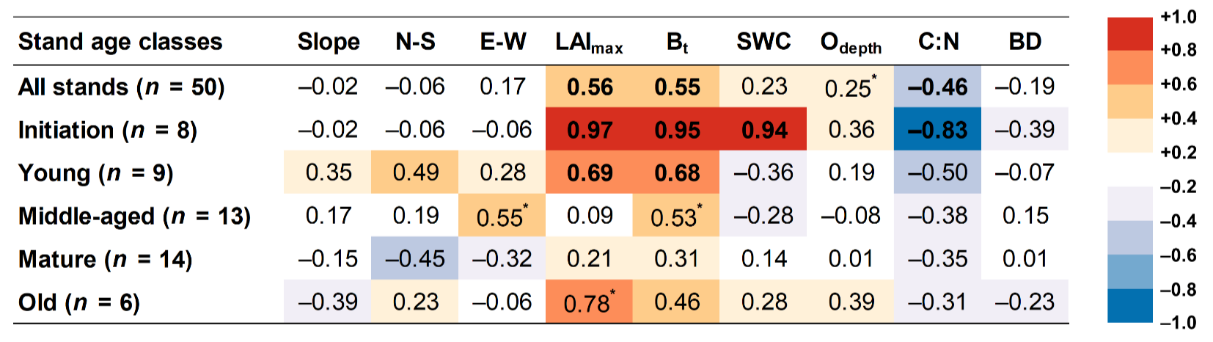


***Table S4.* Heat map based on correlation matrix among biotic and abiotic factors controlling the total heterotrophic respiration (RH).** Warm colours represent positive correlation and cool colours represent negative correlation based on Pearson’s correlation test. Data pooled for all stands and each of the stand age classes (initiation, young, middle-aged, mature, and old). Significant correlations (*p* < 0.05) are marked in bold. Marginally significant correlations (*p* < 0.1) are marked with *. SOC: soil organic carbon up to 20 cm depth (Mg C ha^–1^), O_depth_: depth of the soil organic layer (cm), L: annual detritus production via litterfall (g C m^–2^ yr^–1^), C:N: average soil carbon-nitrogen ratio up to 20 cm depth (dimensionless), Ts: average annual soil temperature at 10 cm depth (°C), SWC: average annual soil volumetric water content at 5 cm depth (%). *n* = 50 main forest stands.

**
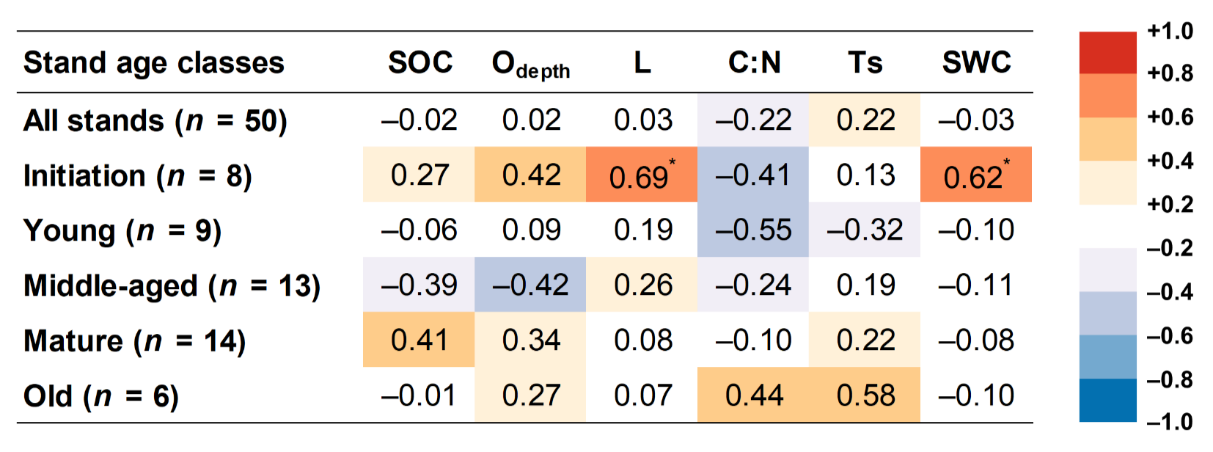
**

**Supplementary methods**

**Section 1. Estimation of net primary production of trees (NPP_t_) for the fifty main forest stands**

An inventory plot (10 m radius) within each of the 50 main forest stands belonging to the regular grid (350×350m) of 556 permanent forest inventory plots spanning the Krycklan Catchment Study (KCS, Laudon et al., 2021) was used for biometric-based measurements of ecosystem net primary production (NPP) and its components. Within a subset of the 47 oldest forest stands, location, species, diameter at breast height (DBH, 1.3 m, ≥ 3 cm), and height (H, ≥ 1.3 m) for all living trees and standing dead trees (assigned to decay class I, (Sandström et al., 2007)) were recorded for each plot from successive forest inventories carried out in April in 2016 and 2018. Missing DBHs in April 2017 and 2019 were derived from increment cores obtained from 150 trees (2–4 representative trees per inventory plot) over the 47 forest stands in November 2020. In addition, downed dead trees were also identified by species, measured for diameter and length, and assigned to a decay class (I−III, Sandström et al., 2007) in October 2019. Annual aboveground and coarse root biomass of living trees (AGB_t_ and BGB_t-cr_, respectively) and standing dead trees (AGB_dw-s_ and BGB_dw-s_, respectively) were estimated for the 47 forest stands during 2016−2018 using species-specific allometric equations as a function of DBH and H (Marklund, 1988; Petersson & Ståhl, 2006; Repola, 2008). Annual total biomass of downed dead trees (B_dw–d_) was also computed for the 47 forest stands over the 3-year study period by multiplying their volume with their species- and decay class-specific wood densities (Sandström et al., 2007). In addition, annual AGB_t_ and BGB_t-cr_ in April 2016, April 2017, April 2018, and April 2019 for the 3 recent clear-cuts (5–7 years-old) included in the 50 forest stand were estimated based on the linear regressions between these biomass components with stand age, which were obtained from the remaining forest stands within the initiation age class (Eq. (1), Table S4).

$\mathrm{AGB}_{t},\mathrm{BGB}_{t-cr}=b_{1}\times Age$ Eq. (1)

Then, dry biomass of standing and downed dead trees was converted to annual carbon (C) stocks (AGC_dw-s_, BGC_dw-s_, and C_dw–d_) by using species-specific C concentrations for each decay class (Mäkinen et al., 2006; Sandström et al., 2007), whereas a C concentration of 50% was assumed to obtain the annual C stocks of living trees (AGC_t_ and BGC_t-cr_).

Annual detritus production via litterfall (L) was also measured for the 47 forest stands during the 3-year period 2016−2018 using three systematically established circular funnel-shaped litter traps (area 0.25 m^2^) per inventory plot, which were positioned 1 m above the ground-level. Plant material was collected at the beginning of the snow-free period (i.e., mid-May) and at monthly intervals thereafter from August to November, oven-dried (60 °C, 48h), sorted into fractions (foliage, branches (≤ 1cm), cones, and miscellaneous), and weighted. The carbon content of dry litter biomass was also assumed to be 50%. For the 3 recent clear-cuts, annual L rates in 2016, 2017, and 2018 were estimated from the relationships between L with stand age, which were defined from the remaining forest stands within the initiation age class (Eq. (2), Table S5).

$L=b_{1}\times\mathrm{Age}^{2}$ Eq. (2)

Annual aboveground net primary production of trees (ANPP_t_, g C m^–2^ yr^–1^; Eq. (3)) was computed in the 50 main forest stands during 2016−2018 as the sum of the annual increment in both aboveground tree and dead wood C stocks (ΔAGC_t_ and ΔAGC_dw–s_, respectively) and annual litterfall production (L). In addition, annual belowground net primary production of coarse roots of trees (BNPP_t-cr_, g C m^–2^ yr^–1^) was computed as the sum of the annual increment in both belowground tree and dead wood C stocks (ΔBGC_t_ and ΔBGC_dw–s_, respectively) according to Eq. (4).

$\mathrm{ANPP}_{t}={\Delta AGC}_{t}+{\Delta AGC}_{dw-s}+L$ Eq. (3)

$\mathrm{BNPP}_{t-cr}={\Delta BGC}_{t}+{\Delta BGC}_{dw-s}$ Eq. (4)

Annual belowground net primary production of fine roots of trees (BNPP_t-fr_, g C m^–2^ yr^–1^) was quantified in the 50 main forest stands over the 3-year study period using the ingrowth core method (Neill, 1992; Vogt et al., 1998) and described in detail in Martínez-García et al. (2022). Then, BNPP_t-fr_ was added to BNPP_t-cr_ to obtain the annual belowground net primary production of trees (BNPP_t_, g C m^–2^ yr^–1^) during 2016−2018 (Eq. (5)).

$\mathrm{BNPP}_{t}=\mathrm{BNPP}_{t-cr}+\mathrm{BNPP}_{t-fr}$ Eq. (5)

Finally, annual net primary production of trees (NPP_t_, g C m^–2^ yr^–1^) in the 50 main forest stands over the 3-year study period was determined as the sum of ANPP_t_ and BNPP_t_ (Eq. (6)).

$\mathrm{NPP}_{t}=\mathrm{ANPP}_{t}+\mathrm{BNPP}_{t}$ Eq. (6)

***Table S5*. Coefficients (standard error in brackets) and statistics for the goodness-of-fit of the regressions used for estimating the above- and belowground tree biomasses (AGB_t_ and BGB_t-cr_, respectively; Mg ha^–1^) in April 2016–2019 using the stand age (Age, yr) as predictive variable.** Equation form is described in Eq. (1). Adjusted coefficient of determination (Adj. R^2^) and standard error of estimate (SEE) of the regressions are shown. Regressions are fitted without intercept (b_0_ = 0) and statistically significant (*p* < 0.05). *n* = 4 main forest stands within the initiation age class (1 forest stand was excluded).

| **Biomass component** | **Year** | **b_1_** | **Adj. R^2^** | **SEE** |
| --- | --- | --- | --- | --- |
| AGB_t_ | April 2016 | 0.316 (0.016) | 0.98 | 0.77 |
|  | April 2017 | 0.358 (0.011) | 0.99 | 0.53 |
|  | April 2018 | 0.377 (0.010) | 0.99 | 0.54 |
|  | April 2019 | 0.385 (0.010) | 0.99 | 0.43 |
|  |  |  |  |  |
| BGB_t-cr_ | April 2016 | 0.090 (0.004) | 0.99 | 0.17 |
|  | April 2017 | 0.102 (0.002) | 0.99 | 0.12 |
|  | April 2018 | 0.108 (0.003) | 0.99 | 0.15 |
|  | April 2019 | 0.110 (0.003) | 0.99 | 0.14 |

***Table S6*. Coefficients (standard error in brackets) and statistics for the goodness-of-fit of the regressions used for estimating the annual litterfall (L, g m^–2^ yr^–1^) using the stand age (Age, yr) as predictive variable.** Equation form is described in Eq. (2). Adjusted coefficient of determination (Adj. R^2^) and standard error of estimate (SEE) of the regressions are shown. Regressions are fitted without intercept (b_0_ = 0) and statistically significant (*p* < 0.05). *n* = 4 main forest stands within the initiation age class (1 forest stand was excluded).

| **Year** | **b_1_** | **Adj. R^2^** | **SEE** |
| --- | --- | --- | --- |
| 2016 | 0.093 (0.018) | 0.82 | 20.23 |
| 2017 | 0.084 (0.013) | 0.87 | 16.82 |
| 2018 | 0.052 (0.014) | 0.69 | 18.95 |

**Section 2. Estimation of net ecosystem production (NEP) for the seven additional old forest stands**

***2.1. Selected forest stands***

Seven additional forest stands within the old stand age class (ranging in age from 169 to 202 years) were selected from the KCS’s plot-network (see Fig. 1 in the manuscript). As for the 50 main forest stands, the inventory plot (10 m radius) belonging to this network was used for biometric- and chamber-based flux measurements. Each forest stand was also classified according to soil type (sediment and till, *n* = 3 and 4, respectively) and dominant tree species (pine and spruce, *n* = 3 and 4, respectively).

***2.2. Net primary production (NPP)***

Similarly to the subset of 47 main forest stands (see section 1), we recorded location, species, diameter at breast height (DBH, 1.3 m, ≥ 3 cm), and height (H, ≥ 1.3 m) for all living trees and standing dead trees (assigned to decay class I, (Sandström et al., 2007)) for the inventory plot of each additional old class forest stand from successive forest inventories carried out in April in 2015 and April 2020. Data from increment cores obtained from 150 trees over the subset of 47 main forest stands in November 2020 (see Section 1) were used to derive the missing DBHs in April 2016, April 2017, April 2018, and April 2019. For this purpose, we obtained the relative annual diameter increment (RDI_i_, %; subscript *i* indicates the study year 2015, …, 2019) from the annual and cumulative ring-width increments obtained over the 5-year period 2015−2019 (RWI_i_ and RWI_15-19_, respectively, mm; Eq. (7)).

${RDI}_{i}={{RWI}_{i}}/{{RWI}_{15-19}}$ Eq. (7)

Then, we computed the annual averaged RDI during 2015−2019 for each tree species and 2.5-cm interval diametric classes. Afterwards, we derived the DBH and H in April of each study year (subscript *i*) for each tree within each additional old class forest stand according to Eqs. (8) and (9).

${DBH}_{i}={DBH}_{15}+\left( {RDI}_{i}\times\left( {DBH}_{20}-{DBH}_{15} \right) \right)$ Eq. (8)

$H_{i}=H_{15}+\left( {RDI}_{i}\times\left( H_{20}-H_{15} \right) \right)$ Eq. (9)

By using above-mentioned species-specific allometric equations based on tree DBH and H (Marklund, 1988; Petersson & Ståhl, 2006; Repola, 2008), the annual aboveground and coarse root biomass of living trees (AGB_t_ and BGB_t-cr_, respectively) and standing dead trees (AGB_dw-s_ and BGB_dw-s_, respectively) were estimated for each additional old class forest stand during the period 2016−2018. Then, dry biomass of living trees was converted to annual carbon (C) stocks (AGC_t_ and BGC_t-cr_) assuming a C content of 50%, whereas dry biomass of standing dead trees was converted to annual carbon (C) stocks (AGC_dw-s_ and BGC_dw-s_) by using species-specific C concentrations for each decay class (Mäkinen et al., 2006; Sandström et al., 2007).

Annual litterfall production (L, g C m^–2^ yr^–1^) was estimated during 2016−2018 based on the relationship between L and the annual net aboveground tree C stock increment (∆AGC_t-net_ = ∆AGC_t_ + ∆AGC_dw-s_, g C m^–2^ yr^–1^). Thus, the annual ratio of L to ∆AGC_t-net_ (L:∆AGC_t-net_, dimensionless) was estimated from the regressions between L:∆AGC_t-net_ and stand basal area (BA, m^2^ ha^−1^) obtained from the 50 main forest stands for each study year (Eqs. (10) and (11), Table S6).

${L:\Delta AGC}_{t-net}=exp\left( b_{0}+b_{1}\times ln\left( \mathrm{BA} \right) \right)$ (2016 and 2018) Eq. (10)

${L:\Delta AGC}_{t-net}=b_{0}+b_{1}\times ln\left( \mathrm{BA} \right)$ (2017) Eq. (11)

Afterwards, we derived the annual L during 2016-2018 based on annual ∆AGC_t-net_ estimates of each additional old class forest stand according to Eq. (10).

$L={\Delta AGC}_{t-net}\times{L:\Delta AGC}_{t-net}$ Eq. (10)

Annual aboveground and belowground coarse root production of trees (ANPP_t_ and BNPP_t-cr_, respectively; g C m^–2^ yr^–1^) were computed during 2016−2018 using a similar approach to that used for the 50 main forest stands (Eqs. (13) and (14)).

$\mathrm{ANPP}_{t}={\Delta AGC}_{t}+{\Delta AGC}_{dw-s}+L$ Eq. (13)

$\mathrm{BNPP}_{t-cr}={\Delta BGC}_{t}+{\Delta BGC}_{dw-s}$ Eq. (14)

Annual aboveground net primary production of understory (ANPP_u_, g C m^–2^ yr^–1^) was estimated from the regressions between ANPP_u_ and stand basal area (BA, m^2^ ha^−1^) obtained from the 50 main forest stands for each study year (Eq. (15), Table S7).

$\mathrm{ANPP}_{u}=exp\left( b_{0}+b_{1}\times\mathrm{BA}^{2} \right)$ Eq. (15)

We then estimated the belowground fine root (diameter ≤ 2 mm) production. Thus, the annual total and understory production (BNPP_fr_ and BNPP_u_, respectively; g C m^–2^ yr^–1^) were obtained from the regressions between BNPP_fr_ and BNPP_u_ versus stand basal area (BA, m^2^ ha^−1^), which were defined from the 50 main forest stands for each study year (Eqs. (16) and (17), respectively; Tables S8 and S9).

$\mathrm{BNPP}_{\mathrm{fr}}=exp\left( b_{0}+b_{1}\times BA \right)$ Eq. (16)

$\mathrm{BNPP}_{u}=exp\left( b_{0}+b_{1}\times BA \right)$ Eq. (17)

Afterwards, the annual belowground fine root production of trees (BNPP_fr-t_, g C m^–2^ yr^–1^) was determined over the 3-year study period from the difference between BNPP_fr_ and BNPP_u_ according to Eq. (18).

$\mathrm{BNPP}_{fr-t}=\mathrm{BNPP}_{\mathrm{fr}}-\mathrm{BNPP}_{u}$ Eq. (18)

Over the 3-year period 2016−2018, annual net primary production of understory (NPP_u_, g C m^–2^ yr^–1^_,_) was calculated as the sum of ANPP_u_ and BNPP_u_ (Eq. (19)), whereas annual net primary production of trees (NPP_t_, g C m^–2^ yr^–1^) was calculated as the sum of ANPP_t_, BNPP_t-cr_, and BNPP_t-fr_ (Eq. (20)). Annual ecosystem net primary production (NPP, g C m^–2^ yr^–1^) was then calculated for each additional old class forest stand as the sum of NPP_u_ and NPP_t_ (Eq. (21)).

$\mathrm{NPP}_{u}=\mathrm{ANPP}_{u}+\mathrm{BNPP}_{u}$ Eq. (19)

$\mathrm{NPP}_{t}=\mathrm{ANPP}_{t}+\mathrm{BNPP}_{t\_cr}+\mathrm{BNPP}_{t\_fr}$ Eq. (20)

$NPP=\mathrm{NPP}_{t}+\mathrm{NPP}_{u}$ Eq. (21)

***2.3. Heterotrophic respiration (RH)***

Annual heterotrophic dead wood respiration of standing dead trees (RH_dw-s_, g C m^–2^ yr^–1^) was derived for each additional old class forest stand by multiplying the annual AGCd_w-s_ and BGC_dw-s_ stocks in 2016, 2017, and 2018 with their decomposition rate constants (k_dw_, yr^−1^). Species-specific k_dw_ rates for each component and decay class were obtained following Shorohova et al. (2008) and Yatskov et al. (2003).

Annual total C stock of downed dead trees (C_dw–d_, Mg C ha^–1^) during 2016–2018 was estimated for each additional old class forest stand from the regressions between C_dw–d_ and total C stock of standing dead trees (C_dw–s_, AGCd_w-s_ + BGC_dw-s_; Mg C ha^–1^) obtained from the 50 main forest stands for each study year (Eq. (22), Table S10).

$C_{dw-d}=b_{0}+b_{1}\times C_{dw-s}$ Eq. (22)

Annual heterotrophic respiration of downed dead trees (RH_dw-d_, g C m^–2^ yr^–1^) during 2016–2018 was then estimated for each additional old class forest stand from the regressions between C_dw–d_ and RH_dw-d_ obtained from the 50 main forest stands for each study year (Eq. (23), Table S11).

$\mathrm{RH}_{dw-d}=b_{0}+b_{1}\times C_{dw-d}$ Eq. (23)

Annual heterotrophic dead wood respiration (RH_dw_, g C m^–2^ yr^–1^) over the 3-year study period was then calculated for each additional old class forest stand as the sum of RH_dw-s_ and RH_dw-d_ (Eq. (24)).

$\mathrm{RH}_{\mathrm{dw}}=\mathrm{RH}_{dw-s}+\mathrm{RH}_{dw-d}$ Eq. (24)

We then estimated the annual heterotrophic soil respiration (RH_s_) for each additional old class forest stand during 2016–2018. As a first step, the soil organic carbon up to 20 cm depth (SOC, g C m^−2^; Eq. (25)) was estimated for each additional old class forest stand from soil samples obtained from sequential cores, which provided C mass fraction (‰), depth of the organic and upper mineral soil (H_depth_, cm), soil bulk density (BD, g cm^−3^) volumetric fraction of coarse inorganic fragments > 2 mm (CRF, %). We assumed a negligible change in SOC at each additional old class forest stand during 2016−2018.

$SOC=\left[ \left( \frac{C}{1000} \right)\times\left( \frac{H_{\mathrm{depth}}}{100} \right)\times BD\times\left( \frac{100-CRF}{100} \right) \right]$ Eq. (25)

The soil organic carbon turnover (τ_s_, yr), which is calculated by dividing SOC (g C m^−2^) by the annual RH_s_ (g C·m^−2^·yr^−1^) (Carvalhais et al., 2014; Chen et al., 2013; Varney et al., 2020), was then estimated from the regressions between τ_s_ and stand basal area (BA, m^2^ ha^−1^) obtained from the 50 main forest stands for each study year (Eq. (26), Table S12).

$\tau_{s}=exp\left( b_{o}+b_{1}\times BA \right)$ Eq. (26)

Afterwards, we derived the annual RH_s_ (g C m^–2^ yr^–1^) during 2016-2018 based on SOC estimates of each additional old class forest stand according to Eq. (27).

$\mathrm{RH}_{s}=\mathrm{SOC}/{\tau_{s}}$ Eq. (27)

Finally, RH_dw_ was added to RH_s_ to obtain the annual total heterotrophic respiration (RH, g C m^–2^ yr^–1^; Eq. (28)).

$RH=\mathrm{RH}_{s}+\mathrm{RH}_{\mathrm{dw}}$ Eq. (28)

***2.4. Net ecosystem production (NEP)***

Annual net ecosystem production (NEP) was determined over the 3-year study period for each additional old class forest stand from the difference between ecosystem NPP and RH (Eq. (29)).

$NEP=NPP-RH$ Eq. (29)

***Table S7*. Coefficients (standard error in brackets) and statistics for the goodness-of-fit of the regressions used for estimating the ratio of litterfall to net aboveground tree C stock increment (L:∆AGC_t-net_, dimensionless) using the stand basal area (BA, m^2^ ha^−1^) as predictive variable.** Equation forms are described in Eqs. (10) and (11). Adjusted coefficient of determination (Adj. R^2^) and standard error of estimate (SEE) of the regressions are shown. Regressions are statistically significant (*p* < 0.05). *n* = 50 main forest stands.

| **Year** | **b_0_** | **b_1_** | **Adj. R^2^** | **SEE** |
| --- | --- | --- | --- | --- |
| 2016 | –2.40 (0.21) | 0.68 (0.07) | 0.65 | 0.51 |
| 2017 | 0.11 (0.16) | 0.21 (0.05) | 0.23 | 0.36 |
| 2018 | –2.41 (0.28) | 0.63 (0.10) | 0.46 | 0.60 |

***Table S8*. Coefficients (standard error in brackets) and statistics for the goodness-of-fit of the regressions used for estimating the aboveground net primary production of understory (ANPP_u_, g C m^−2^ yr^−1^) using the stand basal area (BA, m^2^ ha^−1^) as predictive variable.** Equation form is described in Eq. (15). Adjusted coefficient of determination (Adj. R^2^) and standard error of estimate (SEE) of the regressions are shown. Regressions are statistically significant (*p* < 0.05). *n* = 50 main forest stands.

| **Year** | **b_0_** | **b_1_** | **Adj. R^2^** | **SEE** |
| --- | --- | --- | --- | --- |
| 2016 | 4.29 (0.06) | –0.0006 (0.00008) | 0.51 | 0.29 |
| 2017 | 4.25 (0.06) | –0.0006 (0.00009) | 0.48 | 0.30 |
| 2018 | 3.95 (0.07) | –0.0006 (0.00009) | 0.50 | 0.31 |

***Table S9*. Coefficients (standard error in brackets) and statistics for the goodness-of-fit of the regressions used for estimating the total belowground fine root production (BNPP_fr_, g C m^−2^ yr^−1^) using the stand basal area (BA, m^2^ ha^−1^) as predictive variable.** Equation form is described in Eq. (16). Adjusted coefficient of determination (Adj. R^2^) and standard error of estimate (SEE) of the regressions are shown. Regressions are statistically significant (*p* < 0.05). *n* = 50 main forest stands.

| **Year** | **b_0_** | **b_1_** | **Adj. R^2^** | **SEE** |
| --- | --- | --- | --- | --- |
| 2016 | –1.19 (0.15) | –0.025 (0.006) | 0.23 | 0.50 |
| 2017 | –1.30 (0.16) | –0.024 (0.007) | 0.19 | 0.52 |
| 2018 | –1.64 (0.12) | –0.013 (0.005) | 0.10 | 0.39 |

***Table S10*. Coefficients (standard error in brackets) and statistics for the goodness-of-fit of the regressions used for estimating the belowground fine root production of understory (BNPP_u_, g C m^−2^ yr^−1^) using the stand basal area (BA, m^2^ ha^−1^) as predictive variable.** Equation form is described in Eq. (17). Adjusted coefficient of determination (Adj. R^2^) and standard error of estimate (SEE) of the regressions are shown. Regressions are statistically significant (*p* < 0.05). *n* = 50 main forest stands.

| **Year** | **b_0_** | **b_1_** | **Adj. R^2^** | **SEE** |
| --- | --- | --- | --- | --- |
| 2016 | –1.30 (0.16) | –0.044 (0.007) | 0.46 | 0.53 |
| 2017 | –1.44 (0.17) | –0.042 (0.007) | 0.41 | 0.55 |
| 2018 | –1.91 (0.18) | –0.031 (0.008) | 0.23 | 0.60 |

***Table S11*. Coefficients (standard error in brackets) and statistics for the goodness-of-fit of the regressions used for estimating the total C stock of downed dead trees (C_dw-d_, Mg C ha^−1^) using the total C stock of standing dead trees (C_dw-s_, Mg C ha^−1^) as predictive variable.** Equation form is described in Eq. (22). Data were analysed using a forward stepwise multiple linear regression (MLR) analysis, where year (i.e., 2016, 2017, and 2018) was included as dummy variable. Adjusted coefficient of determination (Adj. R^2^) and standard error of estimate (SEE) of the regressions are shown. Regressions are statistically significant (*p* < 0.05). *n* = 50 main forest stands.

| **Year** | **b_0_** | **b_1_** | **Adj. R^2^** | **SEE** |
| --- | --- | --- | --- | --- |
| 2016, 2017 | 0.21 (0.06) | 0.19 (0.02) | 0.32 | 0.60 |
| 2018 | 0.21 (0.06) | 0.11 (0.03) |  |  |

***Table S12*. Coefficients (standard error in brackets) and statistics for the goodness-of-fit of the regressions used for estimating the heterotrophic respiration of downed dead trees (RH_dw-d_, g C m^−2^ yr^−1^) using the total C stock of downed dead trees (C_dw-d_, Mg C ha^−1^) as predictive variable.** Equation form is described in Eq. (23). Data were analysed using a forward stepwise multiple linear regression (MLR) analysis, where year (i.e., 2016, 2017, and 2018) was included as dummy variable. Adjusted coefficient of determination (Adj. R^2^) and standard error of estimate (SEE) of the regression are shown. Regression is statistically significant (*p* < 0.05). *n* = 50 main forest stands.

| **Year** | **b_0_** | **b_1_** | **Adj. R^2^** | **SEE** |
| --- | --- | --- | --- | --- |
| 2016, 2017, 2018 | 0.05 (0.04) | 3.62 (0.05) | 0.98 | 0.40 |

***Table S13*. Coefficients (standard error in brackets) and statistics for the goodness-of-fit of the regressions used for estimating the effective turnover time for soil carbon (τ_s_ = SOC/RH_s_, yr) using the stand basal area (BA, m^2^ ha^−1^) as predictive variable.** Equation form is described in Eq. (26). Adjusted coefficient of determination (Adj. R^2^) and standard error of estimate (SEE) of the regressions are shown. Regressions are statistically significant (*p* < 0.05). *n* = 50 main forest stands.

| **Year** | **b_0_** | **b_1_** | **Adj. R^2^** | **SEE** |
| --- | --- | --- | --- | --- |
| 2016 | –2.18 (0.10) | 0.014 (0.004) | 0.15 | 0.34 |
| 2017 | –2.11 (0.10) | 0.015 (0.004) | 0.17 | 0.34 |
| 2018 | –2.25 (0.10) | 0.013 (0.004) | 0.13 | 0.34 |

**Section 3. Estimation of leaf area index at peak growing season (LAI_max_) for the remaining twenty five forest stands.**

The leaf area index at peak growing season (LAI_max_, m^2^ m^–2^) was directly quantified in August 2017 in a subset of 25 forest stands using a LAI-2200 plant canopy analyser (Li-Cor Inc., Lincoln, NE, USA) as described by Aguinaga-Gil (2018). LAI_max_ was then estimated for the remaining 25 forest stands from the regressions between LAI_max_ and annual aboveground biomass of living trees (AGB_t_, Mg ha^–1^) defined for both pine- and spruce-dominated stands (Eq. (30), Table S13).

$\mathrm{LAI}_{\max}=b_{1}\times sqrt\left( \mathrm{AGB}_{t} \right)$ Eq. (30)

***Table S14*. Coefficients (standard error in brackets) and statistics for the goodness-of-fit of the regressions used for estimating the leaf area index at peak growing season (LAI_max_, m^2^ m^–2^) in both pine- and spruce-dominated stands using the annual aboveground biomass of living trees (AGB_t_, Mg ha^−1^) as predictive variable.** Equation form is described in Eq. (30). Adjusted coefficient of determination (Adj. R^2^) and standard error of estimate (SEE) of the regressions are shown. Regressions are fitted without intercept (b_0_ = 0) and statistically significant (*p* < 0.05). *n* = 25 main forest stands.

| **Dominant tree species** | **b_1_** | **Adj. R^2^** | **SEE** |
| --- | --- | --- | --- |
| Pine | 0.27 (0.02) | 0.90 | 0.86 |
| Spruce | 0.38 (0.02) | 0.99 | 0.52 |

**References**

Aguinaga-Gil, I. (2018). *Understory above- and belowground biomass allocation over the growing season and across different boreal forest stands in Northern Sweden: A boreal forest landscape study.* (MSc Thesis), Wageningen University and Research (WUR), Wageningen, The Netherlands.

Carvalhais, N., Forkel, M., Khomik, M., Bellarby, J., Jung, M., Migliavacca, M., . . . Reichstein, M. (2014). Global covariation of carbon turnover times with climate in terrestrial ecosystems. *Nature, 514*(7521), 213-217. doi:https://doi.org/10.1038/nature13731

Chen, S., Huang, Y., Zou, J., & Shi, Y. (2013). Mean residence time of global topsoil organic carbon depends on temperature, precipitation and soil nitrogen. *Global and Planetary Change, 100*, 99-108. doi:https://doi.org/10.1016/j.gloplacha.2012.10.006

Laudon, H., Hasselquist, E. M., Peichl, M., Lindgren, K., Sponseller, R., Lidman, F., . . . Ågren, A. M. (2021). Northern landscapes in transition: Evidence, approach and ways forward using the Krycklan Catchment Study. *Hydrological Processes, 35*(4), e14170. doi:https://doi.org/10.1002/hyp.14170

Mäkinen, H., Hynynen, J., Siitonen, J., & Sievänen, R. (2006). Predicting the decomposition of Scots pine, Norway spruce, and Birch stems in Finland. *Ecological Applications, 16*(5), 1865-1879. doi:https://doi.org/10.1890/1051-0761(2006)016[1865:PTDOSP]2.0.CO;2

Marklund, L. G. (1988). *Biomass functions pine, spruce and birch in Sweden.* Department of Forest Survey, Swedish University of Agricultural Sciences, Uppsala, Sweden. (Report 45)

Martínez-García, E., Nilsson, M. B., Laudon, H., Lundmark, T., Fransson, J. E. S., Wallerman, J., & Peichl, M. (2022). Overstory dynamics regulate the spatial variability in forest-floor CO_2_ fluxes across a managed boreal forest landscape. *Agricultural and Forest Meteorology, 318*, 108916. doi:https://doi.org/10.1016/j.agrformet.2022.108916

Neill, C. (1992). Comparison of soil coring and ingrowth methods for measuring belowground production. *Ecology, 73*(5), 1918-1921. doi:https://doi.org/10.2307/1940044

Petersson, H., & Ståhl, G. (2006). Functions for below-ground biomass of *Pinus sylvestris*, *Picea abies*, *Betula pendula* and *Betula pubescens* in Sweden. *Scandinavian Journal of Forest Research, 21*(S7), 84-93. doi:https://doi.org/10.1080/14004080500486864

Repola, J. (2008). Biomass equations for birch in Finland. *Silva Fennica, 42*(4), 236.

Sandström, F., Petersson, H., Kruys, N., & Ståhl, G. (2007). Biomass conversion factors (density and carbon concentration) by decay classes for dead wood of *Pinus sylvestris*, *Picea abies* and *Betula* spp. in boreal forests of Sweden. *Forest Ecology and Management, 243*(1), 19-27. doi:https://doi.org/10.1016/j.foreco.2007.01.081

Shorohova, E., Kapitsa, E., & Vanha-Majamaa, I. (2008). Decomposition of stumps 10 years after partial and complete harvesting in a southern boreal forest in Finland. *Canadian Journal of Forest Research, 38*(9), 2414-2421. doi:https://doi.org/10.1139/X08-083

Varney, R. M., Chadburn, S. E., Friedlingstein, P., Burke, E. J., Koven, C. D., Hugelius, G., & Cox, P. M. (2020). A spatial emergent constraint on the sensitivity of soil carbon turnover to global warming. *Nature Communications, 11*(1), 5544. doi:https://doi.org/10.1038/s41467-020-19208-8

Vogt, K. A., Vogt, D. J., & Bloomfield, J. (1998). Analysis of some direct and indirect methods for estimating root biomass and production of forests at an ecosystem level. *Plant and Soil, 200*(1), 71-89. doi:https://doi.org/10.1023/A:1004313515294

Yatskov, M., Harmon, M. E., & Krankina, O. N. (2003). A chronosequence of wood decomposition in the boreal forests of Russia. *Canadian Journal of Forest Research, 33*(7), 1211-1226. doi:https://doi.org/10.1139/x03-033
